# A Novel Point-Interval-Valued Wind Speed Prediction System from the Perspective of Mixed-Frequency Data

**DOI:** 10.3390/e28080856

**Published:** 2026-08-01

**Authors:** Lue Li, Yuntian Yang, Jun Long, Xingui Zhang

**Affiliations:** 1 Guangxi Key Laboratory of Big Data in Finance and Economics, Guangxi University of Finance and Economics, Nanning 530003, China; liluegxu@163.com; 2 School of Big Data and Artificial Intelligence, Guangxi University of Finance and Economics, Nanning 530003, China; 3 School of Mathematics and Information Science, Guangxi University, Nanning 530003, China; gdmz_zhang@163.com; 4 School of Business, Guangxi University, Nanning 530003, China; junlonggxu@163.com

**Keywords:** wind speed prediction, point-interval-valued prediction, mixed-frequency data, ensemble learning, multi-objective optimization

## Abstract

Accurate wind speed prediction is crucial for enhancing wind power generation efficiency and ensuring grid stability. While previous research has predominantly focused on point-valued or interval-valued predictions using common-frequency data, these approaches often fail to fully capture the multi-scale variability and uncertainty inherent in wind speed sequences. To address this limitation, this paper introduces a novel ensemble prediction system that incorporates mixed-frequency data with point-interval-valued modeling. The proposed framework systematically incorporates mixed-frequency characteristics by applying Symplectic Geometric Mode Decomposition to jointly denoise high-frequency and low-frequency point-interval-valued components; combining Mixed Data Sampling with artificial intelligence models to generate sub-model predictions and mixed-frequency point-interval-valued results and implementing an improved multi-objective ensemble mechanism using the Multi-Objective Rime optimization algorithm to optimally combine the sub-model outputs. Experimental evaluation using real-world wind speed data from two locations in Nanning, China, demonstrates the system’s superior performance, achieving Point-Interval-Valued Mean Absolute Percentage Error values of 3.7984% and 4.7028%, respectively, and outperforming twenty benchmark models. The results highlight the effectiveness of mixed-frequency data in enhancing point-interval-valued prediction accuracy and provide a robust solution for wind energy applications.

## 1. Introduction

This section introduces the research significance of wind speed prediction, reviews the current research status and existing limitations, summarizes recent advances, and highlights the innovations of this study. Recently, Transformer-based forecasting models have demonstrated remarkable capabilities in capturing long-range temporal dependencies for renewable energy forecasting, while diffusion models and foundation models have further improved forecasting robustness and generalization through probabilistic learning and large-scale pre-training, respectively [1,2]. These advances have significantly promoted the development of intelligent renewable energy forecasting. However, most existing studies focus on point-valued prediction using common-frequency data and do not simultaneously exploit mixed-frequency information and point-interval-valued representations.

Unlike existing hybrid forecasting frameworks, which mainly combine decomposition techniques, deep learning models, and optimization algorithms for common-frequency point-valued or interval-valued prediction, the proposed framework establishes a unified mixed-frequency point-interval-valued prediction paradigm. Its novelty lies in the coordinated modeling of mixed-frequency information, point-interval-valued representations, and multi-objective ensemble optimization within a unified framework. This design enables more effective exploitation of multi-scale temporal information and uncertainty, leading to more comprehensive wind speed prediction.

### 1.1. Research Significance

At present, the rapid advancement of the global economy has significantly increased energy consumption, rendering the energy crisis a critical issue of societal concern. As a sustainable and eco-friendly energy source, wind power has been widely adopted worldwide, showing considerable potential as a key alternative to conventional energy reserves [3]. Precise wind speed prediction is essential for the effective incorporation of wind energy systems into electrical grids, guaranteeing both grid reliability and a consistent energy supply [4].

### 1.2. Research Status and Shortcomings

Wind speed patterns exhibit intrinsic randomness and irregular fluctuations [5], with the complex and non-uniform characteristics of wind speed data substantially influencing energy conversion performance. Consequently, optimizing wind power output and ensuring stable functioning of wind energy installations necessitates accurate wind speed predictions. However, current research in wind speed prediction has primarily concentrated on either point-valued (PV) or interval-valued (IV) approaches. While the PV method estimates a single best value of future wind speed at a given time [6], the IV prediction method provides a range with both upper and lower bounds to account for the potential variation in the projected values. Further studies have extended both point-valued and interval-valued prediction approaches within their respective domains. Current studies in wind speed prediction have predominantly overlooked the crucial role of combining mixed-frequency (MF) data with point-interval-valued (PIV) wind speed information. Although MF data can enhance model prediction accuracy and PIV wind speed data offer a richer informational structure, the potential of incorporating MF data into PIV prediction methodologies remains underexplored. Therefore, effectively leveraging MF data structures to comprehensively improve the accuracy of PIV predictions has emerged as a pressing scientific challenge requiring immediate attention.

### 1.3. Research Work

A combined framework for MF-based PIV wind speed prediction must be developed to address this research gap, expand theoretical approaches, improve wind speed prediction accuracy, and enhance wind energy use efficiency. This methodology shows substantial potential for improving PIV-based wind speed prediction in energy generation systems, while supporting the advancement of sustainable power solutions.

### 1.4. Research Progress in Wind Speed Prediction Based on Decomposition Techniques and Multivariate Fusion

Recent advancements in wind speed prediction have increasingly emphasized the integration of decomposition techniques with multivariate data fusion to address the inherent non-stationarity and complexity of wind speed time series. Decomposition methods break down raw signals into more manageable components, facilitating improved forecasting accuracy when combined with deep learning models and multivariate inputs such as temperature, humidity, and atmospheric pressure. Shu and Chan [7] review fractal analysis in wind speed time series, focusing on fractal dimension and Hurst analysis for chaotic behaviors and long-range dependencies. They highlight fractal methods’ potential in hybrid models with machine learning to reveal self-similarity and scale-invariance, enhancing wind dynamics understanding. Liu and Yang [8] examine AI-driven wind speed forecasting, covering multi-scale decompositions like EMD, VMD, and wavelet transforms. They emphasize hybrid models fusing shallow (ANN, SVM) and deep (CNN, LSTM) networks with Multi-Objective optimizers (NSGA-II, MOPSO) to balance accuracy, stability, and efficiency in handling non-stationary signals. Jiang et al. [9] introduce a framework using VMD for decomposition, GNN for atmospheric variable dependencies, and temporal models (Transformer, MLP, LSTM, GRU). It outperforms Transformer-based benchmarks, but Transformer shows lower accuracy than GRU, indicating limitations in multivariate tasks. Liang et al. [10] analyze decomposition methods for short-term forecasting with LSTM and a hybrid model (SVR, RFR, KNN, PSO, LSTM). Comparing EMD variants, VMD, and SSA, they find SSA best for one-step and VMD for multi-step predictions. Secondary decompositions like SSA-SSA minimize errors, boosting performance.

These studies collectively demonstrate that decomposition techniques and multivariate fusion can effectively improve wind speed prediction accuracy by simplifying complex signals and enriching feature representations. However, most existing studies are primarily designed for common-frequency point-valued forecasting and mainly focus on improving prediction accuracy through hybrid modeling. The coordinated utilization of mixed-frequency information and point-interval-valued representations remains insufficiently explored. This limitation motivates the development of the proposed MF-PIV prediction framework.

### 1.5. Review of the Literature

In current wind speed prediction research, the topics under investigation can be classified into two primary categories based on data characteristics: PV prediction obtained from a singular wind speed temporal sequence, and IV prediction grounded in a sequence of interval-defined wind speed data. Thereafter, drawing upon the data properties of these two classifications, MF methodologies were applied to either PV prediction or IV prediction to address the key technical challenges in wind speed prediction. Regardless of which category of research methods is employed, the fundamental objective remains to resolve wind speed prediction challenges through diverse strategies. This research direction has become a major trend in wind speed prediction research.

#### 1.5.1. Wind Speed Prediction Based on Point-Valued Sequence

PV prediction refers to the deterministic estimation of a target variable at a specific future time based on PV inputs. Its characteristics are primarily reflected in PV inputs and PV outputs [11]. A substantial body of research reveals that PV-based prediction techniques dominate the field. In addition, alternative methodologies such as physical modeling, statistical techniques, and artificial intelligence also make valuable contributions. For example, in the domain of physical modeling, Yan et al. [12] combined PV prediction and historical records with conditional probability and generalized additive models to develop probabilistic forecasts of river levels, thereby improving system reliability and decision-making. Mayer and Yang [13] paired PV prediction with ensemble meteorological data to construct a probabilistic photovoltaic output prediction framework, significantly increasing predictive accuracy. Vahidi and Porté-Agel [14] established a physics-based wake model for wind turbines that integrates PV prediction while accounting for turbulence effects, resulting in substantial improvements in predictive fidelity. Within conventional statistical models, Hu et al. [15] developed a hybrid wind speed prediction framework utilizing variational mode decomposition and an optimized echo state network, integrating PV outputs with a differential evolution algorithm to enhance predictive precision. In AI-based models, Demolli et al. [16] introduced an intelligent modeling approach aimed at wind power prediction, utilizing daily wind statistics to enhance PV forecasting accuracy and generalization capability. Similarly, Yan et al. [17] introduced a composite method incorporating enhanced singular spectrum analysis and deep learning; this approach uses PV prediction for sequential wind speed estimation, yielding significant gains in both precision and robustness.

Subsequently, research utilizing MF data has gained increasing prominence. MF analysis involves incorporating variables observed at different temporal frequencies (e.g., hourly, daily, monthly) into a unified modeling framework. The core challenge and innovation lie not merely in using MF data, but in developing methods that can effectively harmonize and leverage the information embedded within these disparate frequencies. Current MF research is characterized by its focus on adapting and extending existing modeling paradigms to handle frequency mismatch, aiming to exploit high-frequency data’s informational richness to improve forecasts of lower-frequency targets. For instance, the Mixed Data Sampling (MIDAS) approach has been a foundational technique in this domain. Its application is evident in works such as that of Hu et al. [18], who constructed a BiLSTM-MIDAS framework that integrates high-frequency online search data to enhance the prediction accuracy of low-frequency tourism demand. Similarly, Chen et al. [19] and An et al. [20] developed novel MF sampling versions of grey system models, demonstrating how traditional grey models can be extended for MF settings to predict indicators like hard disk drive failures and CO_2_ emissions. Beyond specific techniques, MF research is distinguished by its integration into diverse model architectures. Yang et al. [21] constructed an AI ensemble model for wind speed prediction that incorporates MF data, while Jiang et al. [22] employed an MF VAR model to examine causal relationships between quarterly economic growth and annual carbon emissions. Furthermore, Li et al. [23] proposed a system using fuzzy information granulation with reverse MF modeling. These examples illustrate the distinctive trajectory of MF research: moving beyond simple data inclusion towards the development of specialized methodologies and hybrid architectures explicitly designed to process and learn from multi-scale temporal information, thereby offering a more nuanced capture of underlying data dynamics.

PV prediction is widely utilized in time series prediction tasks, including wind speed prediction. However, PV prediction typically relies on the average trend or pattern of historical data and cannot provide uncertainty information. To address this limitation, further research has emerged on PV-to-IV prediction. For example, Tian and Hao [24] introduced an enhanced carbon price analysis and prediction system, which achieves PV-to-IV prediction based on PV prediction results and the distribution function of carbon price sequences. Jiang et al. [25] proposed a hybrid prediction system that unifies PV-to-IV techniques for short-term wind speed prediction, substantially improving both precision and reliability. Li, Wang, and Li [26] introduced a composite prediction framework employing data preprocessing methodologies and the whale optimization algorithm, synthesizing PV-to-IV prediction approaches to generate precise wind speed predictions. Zeng et al. [27] proposed an ensemble prediction framework for carbon prices using MVMD and attention-LSTM, coupling PV-to-IV methods to achieve high-accuracy multi-step predictions. Yang et al. [28] introduced an innovative ensemble system, merging PV-to-IV prediction techniques for wind speed prediction. Wang et al. [29] developed a hybrid model integrating two-layer decomposition, GRU, and KDE for precise PV-to-IV prediction of offshore wind speed. Wang et al. [30] proposed a multistep framework combining TCN-GRU-Attention with error distribution analysis for accurate short-term PV-to-IV prediction of wave height. Wang et al. [31] introduced a PV-to-IV prediction approach based on LSTM-GRU and KDE for significant wave height prediction.

PV-to-IV prediction can overcome the limitations of PV-based prediction by offering more comprehensive and reliable results. However, balancing PV-to-IV prediction remains a challenge. Over-reliance on PV prediction may neglect uncertainty, while excessive reliance on IV prediction can lead to overly cautious decision-making. To address this issue, subsequent studies have introduced IV prediction utilizing IV wind speed series, where IV time series serve as input and yield IV time series as output.

#### 1.5.2. Wind Speed Prediction Based on Interval-Valued Sequence

IV prediction denotes an approach where both inputs and outputs are represented as intervals. In this framework, an IV sequence consists of paired upper and lower boundaries corresponding to each time point. Both the input and output of interval sequences are intervals rather than single values. For instance, Sun et al. [32] developed an ensemble framework leveraging the Multi-Objective Artificial Hummingbird Algorithm, employing IV prediction techniques (such as optimal distribution modeling and explicit upper-lower bound estimation) to produce an IV prediction derived from the IV wind speed data. Chang et al. [33] presented a flood prediction model guided by multi-objective optimization, adopting upper and lower boundary estimation to carry out IV prediction on IV flood data. Liu et al. [34] introduced a secondary decomposition-based ensemble architecture for IV carbon price prediction, which reduces complexity through secondary decomposition, significantly enhancing both forecast accuracy and robustness. Hou et al. [35] designed a hybrid wind power prediction framework combining improved optimization algorithms to provide both deterministic and IV forecasts. Zheng et al. [36] introduced a hybrid architecture utilizing variational mode decomposition and IV long short-term memory networks for IV prediction of crude oil prices and trading strategy design, improving prediction efficiency. Yang et al. [37] proposed an interpretable two-stage ensemble learning-driven IV prediction system, combining deep learning models with the Temporal Fusion Transformer to perform IV prediction of non-ferrous metal prices. This approach effectively captures market volatility and provides robust decision support. Zhu et al. [38] proposed an IV carbon price prediction approach utilizing BEMD decomposition, XGBoost prediction, and DE optimization, which notably enhanced prediction coverage and precision, thereby providing dependable assistance for decision-making in the carbon market. Tang et al. [39] developed an IV carbon price prediction model utilizing IFVMD decomposition combined with LSTM-SVR ensemble aggregation. Through adaptive mode decomposition and optimized weight amalgamation, this model significantly enhances forecast accuracy and effectively captures the interval characteristics of carbon price fluctuations. Hao et al. [40] introduced an IV wind speed prediction system based on Multi-Objective optimization and model selection, achieving high-accuracy predictions for IV wind speed series, enhancing prediction precision. Liu et al. [41] proposed a Multi-Objective ensemble prediction model for IV carbon price based on MF data and sub-model selection. Although IV prediction can be effectively performed, it neglects the information from point value prediction, resulting in reduced prediction coverage. This makes it difficult to analyze more specific wind speed data, thereby hindering effective decision-making for the current wind speed system.

#### 1.5.3. Key Research Gaps Identified in Existing Studies

In prior research, wind speed prediction has predominantly concentrated on either PV predictions or IV predictions. This singular focus overlooks the practical utility embedded in IV wind speed data and fails to explore the complementary advantages of combining both prediction types. Such methodological constraints may result in inadequate information extraction, thereby compromising prediction precision and diminishing the operational efficiency of wind power systems. Earlier works have employed IV time series, which encapsulate the upper and lower bounds at each temporal instance. While PV time series provide discrete observations at specific moments, IV time series encompass the temporal range and uncertainty associated with each observation. Nonetheless, these approaches still fall short in effectively incorporating the intermediate temporal structure and the corresponding interval bounds within the IV time series. The combination of PIV prediction holds considerable promise for enhancing both the accuracy and efficiency of statistical inference. Although considerable advancements have been achieved in wind speed prediction based on CF data, research utilizing MF data remains at an early stage of development. MF data, especially those with higher temporal resolution, offer a more detailed representation of underlying dynamics and have demonstrated substantial potential to enhance prediction capabilities in various application areas. These findings imply that employing MF data within a composite modeling framework—one that incorporates both PV and IV prediction strategies—may provide a novel and effective approach for addressing existing challenges in wind speed prediction. A synthesis of pertinent studies is outlined in Table 1. Overall, although previous studies have achieved considerable progress in point-valued prediction, interval-valued prediction, and mixed-frequency modeling individually, a unified prediction framework that simultaneously exploits mixed-frequency information and point-interval-valued representations has not yet been established. This research gap provides the primary motivation for developing the proposed MF-PIV prediction framework.

#### 1.5.4. Research Scope and Objectives

To address current research limitations and enhance the efficiency of wind energy utilization, this study proposes a prediction framework that combines MF data with PIV time series. Within this framework, MF refers to employing data sampled at different temporal resolutions for prediction purposes; PV characterizes the intermediate elements within IV sequences, while IV denotes time series defined by upper and lower bounds. This approach offers a flexible and innovative modeling strategy, emphasizing the critical role of MF data and PIV representations in advancing the accuracy and comprehensiveness of wind speed prediction.

This paper introduces a novel ensemble wind speed prediction system aimed at enhancing decision-making in power systems. The proposed framework opens a new avenue for research in wind speed prediction and demonstrates considerable potential for further improvement.

The system is composed of the following three key modules, with the MF and PIV composite prediction module. This component employs five distinct models to predict the upper bound, lower bound, and middle value of PIV wind speed sequences. These include the mixed data sampling (MIDAS) model designed to handle MF inputs, three artificial intelligence-based models—BPNN, ELM, and ENN—as well as the GRU model trained on CF data. The third module is the Multi-Objective ensemble module. A novel ensemble methodology is proposed to simultaneously combine the prediction results of the upper, lower, and middle bounds derived from each individual model, thereby enhancing overall prediction accuracy and robustness. To validate the effectiveness of the proposed system, this study introduces novel evaluation metrics for prediction performance, utilizing real-world wind speed data collected from two locations in Nanning, China—Wuming District and Xingning District. Four comparative experimental groups are subsequently designed and analyzed.

Relative to twenty benchmark models, the proposed prediction system attains PIVMAPE values of 3.7984% and 4.7028%, along with corresponding PIVRMSE values of 0.4447 and 0.5450 at Site A and Site B, respectively. These results demonstrate statistically significant improvements over all comparative methods. The findings clearly highlight the advantages of employing MF-based PIV data as opposed to traditional CF-based approaches, leading to substantially enhanced prediction precision. Accordingly, the proposed system provides a robust and practical solution for wind speed prediction, with promising potential for broad application in renewable energy management.

### 1.6. Research Innovations

This study presents the following key innovations and contributions.

(1) This study presents a point-interval-valued (PIV) prediction model to enhance prediction accuracy.

The proposed framework consists of the following: (i) a data preprocessing module (which combines PIV data), (ii) an MF-based prediction module (utilizing PIV prediction techniques), and (iii) a Multi-Objective ensemble module. This system represents a notable technological advancement in wind speed prediction and offers valuable perspectives for upcoming research.

(2) This paper presents mixed-frequency (MF) data and develops a novel wind speed prediction system that incorporates mixed-frequency (MF)-based point-interval-valued (PIV) characteristics.

MF data containing HF and LF information provide more comprehensive features than CF data, enabling models to capture finer details and patterns for improved prediction accuracy. By leveraging MF data, the model better characterizes wind speed dynamics (including volatility and nonlinearity), enhancing prediction reliability and robustness. The incorporation of MF data and PIV features significantly improves the precision and dependability of wind speed predictions.

(3) Tailored for the characteristics of point-interval-valued (PIV) data, this study proposes a novel data preprocessing strategy. This strategy employs the standard Symplectic Geometry Mode Decomposition (SGMD) algorithm to jointly denoise both the low-frequency (LF) and high-frequency (HF) wind speed components within the MF-PIV framework.

A dedicated PIV data preprocessing module is developed. Its key innovation lies in the novel application of SGMD to this specific context: by processing LF and HF sequences in a coordinated manner, it effectively reduces noise interference inherent in raw MF-PIV wind speed data, providing refined inputs for subsequent modeling.

(4) This paper proposes an improved Multi-Objective prediction system incorporating point-interval-valued (PIV) data with adjustable factors.

Based on MF modeling, this study constructs a Multi-Objective ensemble system incorporating adjustable factors. The PIV prediction methods are first embedded into the MF framework, and then all PIV results are integrated through the Multi-Objective Rime optimization algorithm (MORIME) to fully exploit the data potential. With the adjustable factors, the system can obtain better prediction results through optimization, thereby more accurately approaching the true PIV wind speeds. This approach achieves marked accuracy improvements. Experimental results demonstrate its significant advantages in both prediction precision and computational efficiency.

(5) A novel evaluation metric is proposed.

The following six evaluation metrics are introduced for assessing the results: Point-Interval-Valued Mean Absolute Percentage Error (PIVMAPE), Point-Interval-Valued Mean Absolute Error (PIVMAE), Point-Interval-Valued Root Mean Square Error (PIVRMSE), Point-Interval-Valued Theil’s U Statistics (PIVU), Point-Interval-Valued Sum of Squared Error (PIVSSE), and Point-Interval-Valued Mean Relative Variability (PIVARV).

### 1.7. Structure and Design

The structure of the paper is as follows: Section 2 presents the problem and research approach; Section 3 examines the dataset; Section 4 elaborates on the proposed Multi-Objective ensemble system framework and evaluates the outcomes from four experimental groups; Section 5 explores the results and provides further insights; Section 6 summarizes the study.

## 2. Problem and Method

This section elaborates on the problem statement, decomposition techniques for PIV data, CF and MF prediction models, as well as combined optimization algorithms.

### 2.1. Problem Statement

**Figure 1:**
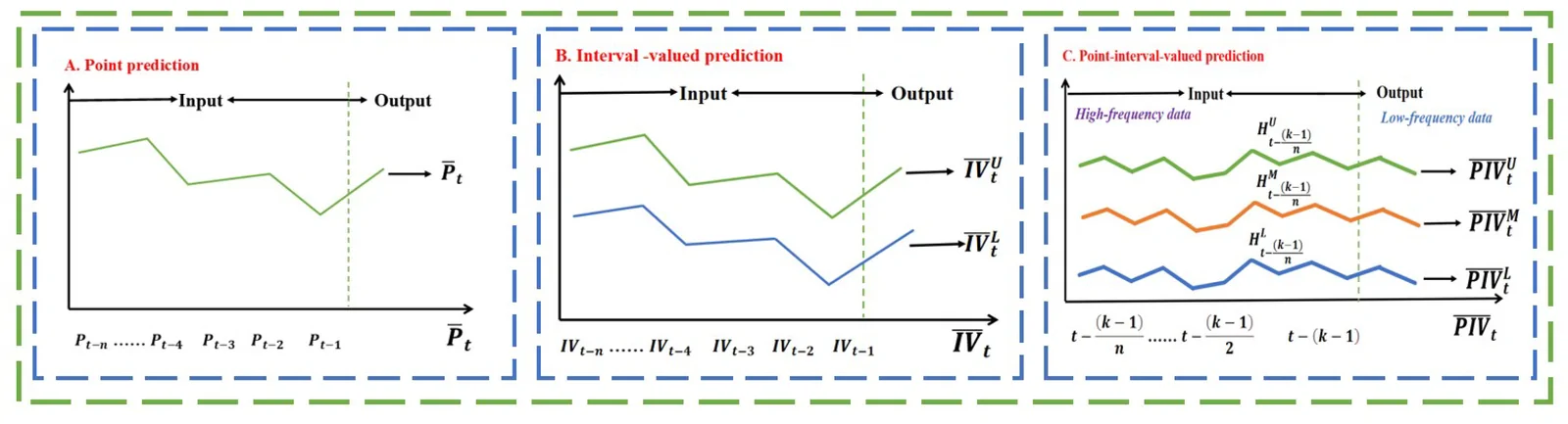
Comparison of three wind speed prediction paradigms: (**A**) point-valued (PV) prediction, (**B**) interval-valued (IV) prediction, and (**C**) mixed-frequency point-interval-valued (MF-PIV) prediction. The proposed MF-PIV paradigm simultaneously utilizes high-frequency and low-frequency information while predicting the upper, middle, and lower bounds of wind speed.

As presented in Figure 1A, PV prediction uses historical data $\left\{P_{t-1},P_{t-2},P_{t-3},\ldots ,P_{t-n}\right\}$ to predict the future value at time *t* and outputs $\left\{{\bar{P}}_{t}\right\}$. However, traditional PV prediction methods depend solely on prediction models, and the resulting predictions may not adequately capture the uncertainty typically observed in wind speed data sequences. As illustrated in Figure 1B, the IV time series can be expressed as

(1) $${\bar{IV}}_{t}=\left\{\left[{\bar{IV}}_{t}^{L},{\bar{IV}}_{t}^{U}\right]:{\bar{IV}}_{t}^{L},{\bar{IV}}_{t}^{U}\in R,\;{\bar{IV}}_{t}^{L}\leq {\bar{IV}}_{t}^{U}\right\},$$

where the lower bound ${\bar{IV}}_{t}^{L}$ and upper bound ${\bar{IV}}_{t}^{U}$ represent the uncertainty in the prediction. The aim is to predict the future value at time *t* and produce the corresponding interval as $\left\{\left[{\bar{IV}}_{t}^{L},{\bar{IV}}_{t}^{U}\right]\right\}$, where ${\bar{IV}}_{t}^{L}$ and ${\bar{IV}}_{t}^{U}$ indicate the lower and upper bounds, respectively. The IV prediction, while capable of providing a prediction range through upper and lower bounds, may overlook local data characteristics when capturing specific information due to data diversity and complexity. As presented in Figure 1C for MF-based PIV data for wind speed prediction, the PIV time series is defined as

(2) $${\bar{PIV}}_{t}=\left\{\left[{\bar{PIV}}_{t}^{L},{\bar{PIV}}_{t}^{M},{\bar{PIV}}_{t}^{U}\right]:{\bar{PIV}}_{t}^{L},{\bar{PIV}}_{t}^{M},{\bar{PIV}}_{t}^{U}\in R,\;{\bar{PIV}}_{t}^{L}\leq {\bar{PIV}}_{t}^{M}\leq {\bar{PIV}}_{t}^{U}\right\}$$

where ${\bar{PIV}}_{t}^{L}$, ${\bar{PIV}}_{t}^{M}$, and ${\bar{PIV}}_{t}^{U}$ represent the lower bound, middle bound, and upper bound, respectively, in the prediction of the wind speed at time *t*. Regarding PIV data, the prediction uses HF historical data

(3) $$\left\{\left[H_{t-(k-1)}^{L},H_{t-(k-1)}^{M},H_{t-(k-1)}^{U}\right],\left[H_{t-\frac{\left(k-1\right)}{2}}^{L},H_{t-\frac{\left(k-1\right)}{2}}^{M},H_{t-\frac{\left(k-1\right)}{2}}^{U}\right],\ldots ,\left[H_{t-\frac{\left(k-1\right)}{n}}^{L},H_{t-\frac{\left(k-1\right)}{n}}^{M},H_{t-\frac{\left(k-1\right)}{n}}^{U}\right]\right\}$$

to determine the upcoming value at time *t*, and finally predicts the LF PIV data $\left\{\left[{\bar{PIV}}_{t}^{L},{\bar{PIV}}_{t}^{M},{\bar{PIV}}_{t}^{U}\right]\right\}$.

At present, wind speed prediction research frequently overlooks the combination of PIV data. The majority of studies predominantly focus on CF data, leading to suboptimal wind energy utilization and a considerable disregard for the potential benefits of MF data.

#### 2.1.1. SGMD

Symplectic Geometric Mode Decomposition (SGMD) is a signal processing technique for decomposing complex signals into a series of Symplectic Geometric Components (SGCs) [44]. It enhances data quality through noise reduction and feature extraction, providing cleaner inputs for models and improving forecasting accuracy [45]. The symplectic geometric framework enables effective decoupling of different frequency components. SGMD is particularly advantageous for LF data, as it captures low-frequency modes without losing information, allowing for accurate recovery of data characteristics and reduction in low-frequency noise. For HF data, SGMD can precisely separate rapidly changing components, effectively extracting key features while retaining detailed signal information and eliminating high-frequency noise interference.

The essential steps of the SGMD algorithm are outlined below:

Step 1: Construct the Trajectory Matrix *X*

According to the embedding theorem, the original time series x(t) can be mapped onto the trajectory matrix *X*, which captures all dynamic information of the time series x(t). The trajectory matrix *X* is defined as follows:

(4) $$X=\left(\begin{array}{cccc} x_{1} & x_{1+s} & \ldots & x_{1+(d-1)s}\\ x_{2} & x_{2+s} & \ldots & x_{2+(d-1)s}\\ \vdots & \vdots & \ddots & \vdots \\ x_{m} & x_{m+s} & \ldots & x_{m+(d-1)s} \end{array}\right)$$

where *d* represents the embedding dimension, *s* denotes the delay time, and m=n−(d−1)s.

Step 2: Compute the Symmetric Covariance Matrix *A*

By performing correlation analysis of the time series on the trajectory matrix *X*, the symmetric covariance matrix *A* is obtained as follows:

(5) $$A=X^{T}X$$

Step 3: Construct the Hamiltonian Matrix *M*

Using the symmetric matrix *A*, the Hamiltonian matrix *M* is constructed as follows:

(6) $$M=\left(\begin{array}{cc} A & 0\\ 0 & -A^{T} \end{array}\right)$$

Step 4: Construct the Symplectic Orthogonal Matrix *P*

In light of the characteristics of the Hamiltonian matrix, the symplectic orthogonal matrix *P* is subsequently derived as follows:

(7) $$P^{T}MP=\left(\begin{array}{cc} B & R\\ 0 & B^{T} \end{array}\right)$$

where *P* is an orthogonal symplectic matrix, and *B* is an upper triangular matrix.

Step 5: Calculate the Eigenvalues and Eigenvectors

The eigenvalues $r_{i}$ corresponding to the upper triangular matrix *B* are computed using the following expression:

(8) $$r_{i}=\sqrt{\lambda_{i}},\;i=1,2,\ldots ,n$$

where $\lambda_{i}$ are the eigenvalues of the Hamiltonian matrix *M*. The eigenvectors $\Pi_{i}$ corresponding to the eigenvalues of *A* are used to construct the matrix *P*.

Step 6: Reconstruct the Signal Components

The reconstructed signal components are obtained through the following steps:

(9) $$S=P^{T}X$$

(10) Z=PS

where *Z* denotes the reconstructed trajectory matrix. Subsequently, diagonal averaging is applied to the initial single-component matrix *Z*, converting it into a new set of time series with length *n*:

(11) $$t_{k}=\left\{\begin{array}{cc} \frac{1}{k}\sum_{p=1}^{k}z_{p,p+1} & \mathrm{if}\;1\leq k<d\\ \frac{1}{d}\sum_{p=1}^{d}z_{p,k-p+1} & \mathrm{if}\;d\leq k\leq m^{*}\\ \frac{1}{n-k+1}\sum_{p=1}^{n-m^{*}+1}z_{p,k-p+1} & \mathrm{if}\;m^{*}<k\leq n \end{array}\right.$$

where $m^{*}=\max\left(m,d\right)$ and n=m+(d−1)s.

Step 7: Compute the Increment of Energy Entropy and the Frequency Cross-Correlation Coefficient

For each SGC component $u_{i}\left(t\right)$, the energy $E(u_{i}\left(t\right))$ is calculated as follows:

(12) $$E\left(u_{i}\left(t\right)\right)=\int_{0}^{T}u_{i}{\left(t\right)}^{2}dt$$

The normalized energy p(i) is then calculated as follows:

(13) $$p\left(i\right)=\frac{E(u_{i}\left(t\right))}{\sum_{i=1}^{N}E\left(u_{i}\left(t\right)\right)}$$

The energy entropy increment Dq is calculated as follows:

(14) Dq=−p(i)log(p(i))

where $E(u_{i}\left(t\right))$ denotes the energy of the *i*-th SGC component, p(i) is the normalized energy ratio of the component, and $D_{q}$ represents the corresponding energy entropy increment. The frequency cross-correlation coefficient $q_{f}$ is calculated as follows:

(15) $$q_{f}=\frac{\int_{0}^{f_{a}}G_{x_{i}}G_{y}df}{\sqrt{\int_{0}^{f_{a}}G_{x_{i}}^{2}df\int_{0}^{f_{a}}G_{y}^{2}df}}$$

where $G_{x_{i}}$ and $G_{y}$ are the power spectra of the signals $x_{i}$ and *y*, respectively, and $f_{a}$ is the analysis frequency.

Step 8: Extract the Effective Components

Based on the energy entropy increment and frequency cross-correlation coefficient, the effective components are selected to reconstruct the signal, achieving the denoising effect.

#### 2.1.2. MIDAS

The MIDAS model incorporates variables sampled at varying frequencies into a unified framework, employing HF data to explicate LF phenomena, thus improving the precision of LF data forecasts. By integrating HF and LF data, the MIDAS model mitigates information loss [46]. It effectively utilizes multi-frequency data and a diverse set of predictor variables [47]. Nevertheless, assuming a generic distributed lag model may lead to parameter dispersion. To address this issue, a weighting scheme is introduced for the HF variable, considering lags up to the maximum order K−1, thereby achieving frequency alignment. The general form of the MIDAS model is given by the following:

(16) $$L_{t}=\alpha_{0}+\alpha_{1}\sum_{k=1}^{K}\omega \left(k;\theta \right)L^{(k-1)/n}H_{t}^{\left(n\right)}+\epsilon_{t}$$

In this equation, $L_{t}$ denotes the LF target variable, and $\alpha_{0}$ is the intercept term. The parameter $\alpha_{1}$ is the coefficient associated with the HF explanatory variable $H_{n}$. The function ω(k;θ) represents a parameterized weight function depending on θ, and the term $L^{(k-1)/n}H_{n}$ applies a fractional lag operator to the HF variable, where *n* is the frequency ratio between HF and LF data. The variable $H_{n}$ is the HF predictor, and $\epsilon_{t}$ is the error term that captures unexplained residual variation.

To specify the lag operator used in the equation, we define the following:

(17) $$L^{(k-1)/n}H_{t}=H_{\left(t-(k-1)/n\right)}^{\left(n\right)}$$

This formulation indicates that $H_{\left(t-(k-1)/n\right)}^{\left(n\right)}$ is the value of the HF variable at the shifted time point corresponding to the (k−1)/n lag.

To account for the potential temporal dependency in the LF target variable, an autoregressive MIDAS model can be employed, expressed as follows:

(18) $$L_{t}=\alpha_{0}+\sum_{q=1}^{Q}\delta_{q}L_{t-q}+\alpha_{1}B\left(L^{1/n};\theta \right)H_{t-h/n}^{n}+\epsilon_{t}$$

In this extended formulation, $\delta_{q}$ are the coefficients for the autoregressive terms of the LF variable, and *Q* denotes the autoregressive order. The function $B(L^{1/n};\theta )$ is a weighting function applied to the lagged HF data. The term $H_{t-h/n}^{n}$ refers to the HF predictor at a future or lagged time point, where *h* is the lead or lag horizon expressed relative to the HF.

To obtain the PIV wind speed, the following key symbols are defined. $\left\{H_{i}|i=1,2,\ldots ,I\right\}$: HF impact variables. $\left\{L_{t}|t=1,2,\ldots ,T\right\}$: LF predictive variables. $\left[H_{t}^{\mathrm{Min}},H_{t}^{\mathrm{Middle}},H_{t}^{\mathrm{Max}}\right]$, respectively, denote the lower, middle, and upper bounds of the HF component. $\left[L_{t}^{\mathrm{Min}},L_{t}^{\mathrm{Middle}},L_{t}^{\mathrm{Max}}\right]$ respectively represent the lower, middle, and upper bounds of the predicted LF component. The middle value represents the mean value over the corresponding time period, indicating IV information. The specific process is as follows:

(19) $$\begin{array}{ccc} \left[\begin{array}{ccc} L_{2}^{\mathrm{Min}} & L_{2}^{\mathrm{Middle}} & L_{2}^{\mathrm{Max}}\\ \vdots & \vdots & \vdots \\ L_{n}^{\mathrm{Min}} & L_{n}^{\mathrm{Middle}} & L_{n}^{\mathrm{Max}} \end{array}\right] & = & \left[\begin{array}{ccc} L_{1}^{\mathrm{Min}} & L_{1}^{\mathrm{Middle}} & L_{1}^{\mathrm{Max}}\\ \vdots & \vdots & \vdots \\ L_{n-1}^{\mathrm{Min}} & L_{n-1}^{\mathrm{Middle}} & L_{n-1}^{\mathrm{Max}} \end{array}\right]\left[\begin{array}{ccc} \alpha_{1} & \alpha_{4} & \alpha_{7}\\ \alpha_{2} & \alpha_{5} & \alpha_{8}\\ \alpha_{3} & \alpha_{6} & \alpha_{9} \end{array}\right]\\ & & +\left[\begin{array}{ccc} H_{1}^{\mathrm{Min}} & \ldots & H_{3}^{\mathrm{Max}}\\ \vdots & \ddots & \vdots \\ H_{n-1}^{\mathrm{Min}} & \ldots & H_{n+1}^{\mathrm{Max}} \end{array}\right]\left[\begin{array}{ccc} \beta_{1} & \beta_{10} & \beta_{19}\\ \vdots & \vdots & \vdots \\ \beta_{9} & \beta_{18} & \beta_{27} \end{array}\right]\\ & & +\left[\begin{array}{ccc} \epsilon_{2}^{\mathrm{Min}} & \epsilon_{2}^{\mathrm{Middle}} & \epsilon_{2}^{\mathrm{Max}}\\ \vdots & \vdots & \vdots \\ \epsilon_{n}^{\mathrm{Min}} & \epsilon_{n}^{\mathrm{Middle}} & \epsilon_{n}^{\mathrm{Max}} \end{array}\right] \end{array}$$

In the illustrative case above, the lag order of HF historical data is d=9, while that of the LF influencing variables is b=3. Accordingly, the generalized matrix expression can be formulated as follows:

(20) $$\begin{array}{ccc} \left[\begin{array}{ccc} L_{b+2}^{\mathrm{Min}} & L_{b+2}^{\mathrm{Middle}} & L_{b+2}^{\mathrm{Max}}\\ L_{b+3}^{\mathrm{Min}} & L_{b+3}^{\mathrm{Middle}} & L_{b+3}^{\mathrm{Max}}\\ \vdots & \vdots & \vdots \\ L_{n}^{\mathrm{Min}} & L_{n}^{\mathrm{Middle}} & L_{n}^{\mathrm{Max}} \end{array}\right] & = & \left[\begin{array}{ccc} L_{b+1}^{\mathrm{Min}} & L_{b+1}^{\mathrm{Middle}} & L_{b+1}^{\mathrm{Max}}\\ L_{b+2}^{\mathrm{Min}} & L_{b+2}^{\mathrm{Middle}} & L_{b+2}^{\mathrm{Max}}\\ \vdots & \vdots & \vdots \\ L_{n-1}^{\mathrm{Min}} & L_{n-1}^{\mathrm{Middle}} & L_{n-1}^{\mathrm{Max}} \end{array}\right]\left[\begin{array}{ccc} \alpha_{1} & \alpha_{b+1} & \alpha_{2b+1}\\ \alpha_{2} & \alpha_{b+2} & \alpha_{2b+2}\\ \vdots & \vdots & \vdots \\ \alpha_{b} & \alpha_{2b} & \alpha_{3b} \end{array}\right]\\ & & +\left[\begin{array}{cccc} H_{m(b+2-h)}^{\mathrm{Min}} & H_{m(b+2-h)+1}^{\mathrm{Middle}} & \ldots & H_{m(b+2-h)+d-1}^{\mathrm{Max}}\\ H_{m(b+3-h)}^{\mathrm{Min}} & H_{m(b+3-h)+1}^{\mathrm{Middle}} & \ldots & H_{m(b+3-h)+d-1}^{\mathrm{Max}}\\ \vdots & \vdots & \ddots & \vdots \\ H_{m(n-h)}^{\mathrm{Min}} & H_{m(n-h)+1}^{\mathrm{Middle}} & \ldots & H_{m(n-h)+d-1}^{\mathrm{Max}} \end{array}\right]\left[\begin{array}{ccc} \beta_{1} & \beta_{d+1} & \beta_{2d+1}\\ \beta_{2} & \beta_{d+2} & \beta_{2d+2}\\ \vdots & \vdots & \vdots \\ \beta_{d} & \beta_{2d} & \beta_{3d} \end{array}\right]\\ & & +\left[\begin{array}{ccc} \epsilon_{b+2}^{\mathrm{Min}} & \epsilon_{b+2}^{\mathrm{Middle}} & \epsilon_{b+2}^{\mathrm{Max}}\\ \epsilon_{b+3}^{\mathrm{Min}} & \epsilon_{b+3}^{\mathrm{Middle}} & \epsilon_{b+3}^{\mathrm{Max}}\\ \vdots & \vdots & \vdots \\ \epsilon_{n}^{\mathrm{Min}} & \epsilon_{n}^{\mathrm{Middle}} & \epsilon_{n}^{\mathrm{Max}} \end{array}\right] \end{array}$$

#### 2.1.3. BPNN

The Back Propagation Neural Network (BPNN) is a widely utilized type of artificial neural network within the domain of supervised learning. It is composed of an input layer, one or more hidden layers, and an output layer. The training process is conducted via the backpropagation algorithm, which adjusts network parameters to minimize the objective function value [48]. A BPNN consists of an input layer, an output layer, and several hidden layers, each fulfilling distinct roles in information processing. The input layer is responsible for receiving data, the hidden layers handle the processing of the data, and the output layer produces the final results. Each layer comprises multiple neurons that contribute to the network’s learning capabilities [49]. The formula is as follows:

(21) $$h_{j}^{\left[l\right]}=\sigma^{\left[l\right]}\left(\sum_{i=1}^{n}x_{i}^{\left[l-1\right]}\cdot w_{ij}^{\left[l\right]}+b_{j}^{\left[l\right]}\right)$$

where $h_{j}^{\left[l\right]}$ represents the output of the *j*-th neuron in the *l*-th hidden layer. $\sigma^{\left[l\right]}$ is the activation function applied in the *l*-th layer. $x_{i}^{\left[l-1\right]}$ is the input from the *i*-th neuron of the previous layer (l−1). $w_{ij}^{\left[l\right]}$ is the weight connecting the *i*-th neuron of the previous layer to the *j*-th neuron of the current layer. $b_{j}^{\left[l\right]}$ is the bias term for the *j*-th neuron in the *l*-th layer.

(22) $$E^{\left[L\right]}=\frac{1}{2}\sum_{j=1}^{m}{\left(T_{j}-{\hat{y}}_{j}\right)}^{2}$$

where $E^{\left[L\right]}$ is the error of the output layer. $T_{j}$ denotes the target value associated with the *j*-th output neuron. ${\hat{y}}_{j}$ indicates the estimated output for the *j*-th output neuron.

(23) $$\Delta w_{ij}^{\left[l\right]}=-\eta \frac{\partial E^{\left[L\right]}}{\partial w_{ij}^{\left[l\right]}}$$

where $\Delta w_{ij}^{\left[l\right]}$ is the change in the weight connecting the *i*-th neuron of layer (l−1) to the *j*-th neuron of layer *l*. η is the learning rate.

(24) $$\Delta b_{j}^{\left[l\right]}=-\eta \frac{\partial E^{\left[L\right]}}{\partial b_{j}^{\left[l\right]}}$$

where $\Delta b_{j}^{\left[l\right]}$ is the change in the bias of the *j*-th neuron in layer *l*. This algorithm is applied in (21).

#### 2.1.4. ELM

ELM is a form of feedforward neural network featuring a single hidden layer. Unlike traditional training approaches for single hidden layer feedforward neural networks, ELM offers multiple benefits, such as improved learning efficiency, shorter training duration, and the random assignment of learning parameters [50]. A key characteristic of the ELM algorithm is that, during the training stage, the parameters for the hidden layer nodes are randomly generated from any continuous probability distribution. The feature mapping can be either randomly produced or explicitly specified. As only the output weights need to be calculated, ELM can be regarded as a linear parameter optimization process, with the learning procedure generally converging to the global optimum [51]. The corresponding formula is as follows:

Step 1: Input to Hidden Layer

(25) z=Wx+b

*x*: Input vector, *W*: Hidden layer weights, *b*: Bias, *z*: Weighted input to the hidden layer.

Step 2: Hidden Layer Output (Activation Function).

(26) h=f(z)

*h*: Output of the hidden layer, f(·): Activation function (e.g., Sigmoid, ReLU).

Step 3: Output Layer

(27) $$\hat{y}=\beta h$$

$\hat{y}$: Predicted output, β: Output layer weights.

Step 4: Training (Solving for Output Layer Weights)

(28) $$\beta ={\left(H^{T}H\right)}^{-1}H^{T}Y$$

*H*: Hidden layer output matrix, *Y*: Target output, β: Output layer weights.

Step 5: Testing Phase

(29) $$\hat{y_{\mathrm{new}}}=\beta \cdot f\left(Wx_{\mathrm{new}}+b\right)$$

$x_{\mathrm{new}}$: New input sample, $\hat{y_{\mathrm{new}}}$: Predicted output for the new sample.

(30) $$f\left(x\right)=\sum_{i=1}^{L}\beta_{i}G\left(a_{i},b_{i},x\right)$$

The ELM model can be used for both classification and regression tasks, and it has been widely applied in various fields due to its simplicity and efficiency. This algorithm is applied in (21).

#### 2.1.5. ENN

ENN embodies a distinctive type of local feedback neural network, which may be understood as a recurrent neural architecture equipped with localized memory elements and feedback loops [52]. In contrast to conventional feedforward neural architectures, this advanced network incorporates an associative layer within the hidden layer, functioning as a delay mechanism for data storage. This configuration facilitates the model in efficiently capturing time-dependent dynamics and substantially bolsters the overall reliability of the prediction system [53].

Step 1: Define Inputs and Weights. Let $X=[x_{1},x_{2},\ldots ,x_{n}]$ be the input vector, and $W=[w_{1},w_{2},\ldots ,w_{n}]$ be the corresponding weight vector. $x_{i}$: i-th input feature; $w_{i}$: weight for i-th input; *n*: input dimension.

Step 2: Compute Weighted Sum

(31) $$Z=\sum_{i=1}^{n}w_{i}x_{i}$$

*Z*: weighted sum/pre-activation.

Step 3: Apply Activation Function

(32) $$Y=f\left(\sum_{i=1}^{n}w_{i}x_{i}\right)$$

f(·): activation function, e.g., sigmoid, ReLU; *Y*: neuron output.

Step 4: Compute Error

(33) $$E=\frac{1}{2}{\left(Y-T\right)}^{2}$$

*T*: target/ground-truth value; *E*: mean squared error loss for one sample.

Step 5: Backpropagate Error and Update Weights

(34) $$w_{i}=w_{i}-\eta \frac{\partial E}{\partial w_{i}}$$

(35) $$\frac{\partial E}{\partial w_{i}}=\left(Y-T\right)\cdot f^{\prime }\left(Z\right)\cdot x_{i}$$

η: learning rate; $f^{\prime }\left(Z\right)$: derivative of activation function at *Z*.

The ENN model adjusts the weights using the backpropagation algorithm to minimize the output error. This error-based self-correction mechanism allows the model to gradually optimize and adapt to different data patterns, thereby improving the model’s accuracy and generalization ability. This algorithm is applied in (21).

#### 2.1.6. GRU

The GRU network is a variant of recurrent neural networks, specifically designed to handle and predict dynamic nonlinear sequences, particularly issues related to long-term dependencies, such as time series data [54]. The GRU controls the storage of information and the updating of sequential data through a dual-port structure, which includes reset and update gates [55]. The mathematical formulation of the GRU mechanism is presented as follows:

Step 1: Update Gate

(36) $$z_{t}=\sigma_{g}\left(W_{z}x_{t}+U_{z}h_{t-1}+b_{z}\right)$$

Here, $z_{t}$ denotes the update gate, and $\sigma_{g}$ represents the activation function. $W_{z}$ and $U_{z}$ are the corresponding weight matrices, $x_{t}$ is the input vector at time *t*, $h_{t-1}$ refers to the hidden state from the previous time step, and $b_{z}$ is the associated bias term.

Step 2: Reset Gate

(37) $$r_{t}=\sigma_{g}\left(W_{r}x_{t}+U_{r}h_{t-1}+b_{r}\right)$$

where $r_{t}$ is the reset gate, $W_{r}$ and $U_{r}$ are weight matrices, and $b_{r}$ is the bias term.

Step 3: Candidate Hidden State

(38) $${\tilde{h}}_{t}=\sigma_{h}\left(W_{h}x_{t}+\left(r_{t}\circ U_{h}h_{t-1}\right)+b_{h}\right)$$

where ${\tilde{h}}_{t}$ represents the candidate hidden state, $\sigma_{h}$ denotes the activation function, $W_{h}$ and $U_{h}$ are the associated weight matrices, ∘ signifies the element-wise (Hadamard) product, and $b_{h}$ corresponds to the bias vector.

Step 4: Final Hidden State

(39) $$h_{t}=z_{t}\circ h_{t-1}+\left(1-z_{t}\right)\circ {\tilde{h}}_{t}$$

where $h_{t}$ is the final hidden state at time step *t*.

The GRU model is less prone to the vanishing gradient problem during training, and the training process is typically more stable. This algorithm is applied in (21).

### 2.2. RIME

The Rime Ice Meta-heuristic algorithm was proposed by [56]. Inspired by the growth dynamics of rime-ice, RIME simulates soft-rime exploration and hard-rime exploitation to balance global search and local refinement. The algorithm proceeds as follows:

Step 1: Initialization

Initialize a population of *N* rime-agents ${\left\{S_{i}\right\}}_{i=1}^{N}$ in a *d*-dimensional space. Each agent $S_{i}$ consists of *d* rime-particles $x_{ij}$, initialized within $\left[{\mathrm{lb}}_{j},{\mathrm{ub}}_{j}\right]$:

(40) $$x_{ij}={\mathrm{lb}}_{j}+\mathrm{rand}\cdot \left({\mathrm{ub}}_{j}-{\mathrm{lb}}_{j}\right),\;i=1,\ldots ,N,\;j=1,\ldots ,d,$$

where rand∼U[0,1]. Here, *N* is the population size, and ${\mathrm{lb}}_{j}$, ${\mathrm{ub}}_{j}$ are the lower and upper bounds of the search space in the *j*-th dimension.

Step 2: Fitness Evaluation

Evaluate the fitness of each agent using the objective function *f*:

(41) $$F\left(S_{i}\right)=f\left(S_{i}\right).$$

Here, $F(S_{i})$ denotes the fitness of agent $S_{i}$.

Step 3: Soft-rime Search Strategy (Exploration)

Model the random motion of soft-rime particles under low wind conditions. Update particle positions via

(42) $$x_{ij}^{\mathrm{new}}=R_{\mathrm{best},j}+r_{1}\cdot \cos\theta \cdot \beta \cdot \left(h\cdot \left({\mathrm{ub}}_{j}-{\mathrm{lb}}_{j}\right)+{\mathrm{lb}}_{j}\right),\;\mathrm{if}\;r_{2}<E,$$

where $r_{1}∼U\left(-1,1\right)$ and $r_{2}∼U\left(0,1\right)$ control movement direction and condensation probability, $\theta =\pi \cdot \frac{t}{10t_{\max}}$ is an angle parameter decreasing with iterations, $\beta =1-\frac{\left\lfloor w\cdot t/t_{\max}\right\rfloor }{w}$ is a stepwise environmental factor with w=5 segments, h∼U(0,1) controls adhesion degree (particle distance), and $E=\sqrt{t/t_{\max}}$ is the attachment coefficient increasing with iterations. Here, *t* is the current iteration and $t_{\max}$ is the maximum number of iterations.

Step 4: Hard-rime Puncture Mechanism (Exploitation)

Simulate directional growth of hard-rime particles under high wind. Perform dimensional crossover with the best agent:

(43) $$x_{ij}^{\mathrm{new}}=R_{\mathrm{best},j},\mathrm{if}\;r_{3}<F^{\mathrm{normr}}\left(S_{i}\right),$$

where

(44) $$F^{\mathrm{normr}}\left(S_{i}\right)=\frac{F\left(S_{i}\right)-F_{\min}}{F_{\max}-F_{\min}+\epsilon },$$

with $\epsilon =10^{-8}$ to avoid division by zero, and $r_{3}∼U\left(0,1\right)$ is a random threshold for crossover. Here, $F_{\min}$ and $F_{\max}$ are the minimal and maximal fitness values in the current population.

Step 5: Positive Greedy Selection Mechanism

Update the population by retaining superior solutions:

(45) $$S_{i}^{\mathrm{new}}=\left\{\begin{array}{cc} \mathrm{Updated}\;\mathrm{agent}\;\mathrm{with}\;\mathrm{new}\;\mathrm{particles}, & \mathrm{if}\;F\left(S_{i}^{\mathrm{new}}\right)<F\left(S_{i}\right),\\ S_{i}, & \mathrm{otherwise}. \end{array}\right.$$

If $F\left(S_{i}^{\mathrm{new}}\right)<F\left(R_{\mathrm{best}}\right)$, update the global best solution by

(46) $$R_{\mathrm{best}}\leftarrow S_{i}^{\mathrm{new}}.$$

Step 6: Boundary Constraint Handling

Enforce search space boundaries for all particles:

(47) $$x_{ij}^{\mathrm{new}}=\left\{\begin{array}{cc} {\mathrm{lb}}_{j}, & \mathrm{if}\;x_{ij}^{\mathrm{new}}<{\mathrm{lb}}_{j},\\ {\mathrm{ub}}_{j}, & \mathrm{if}\;x_{ij}^{\mathrm{new}}>{\mathrm{ub}}_{j},\\ x_{ij}^{\mathrm{new}}, & \mathrm{otherwise}. \end{array}\right.$$

Step 7: Termination Condition

Repeat Steps 2–6 until $t=t_{\max}$ (maximum iterations) or the fitness convergence criterion is met, for example,

(48) $$|F{\left(R_{\mathrm{best}}\right)}^{t}-F{\left(R_{\mathrm{best}}\right)}^{t-1}|<\delta ,$$

where δ is a small positive threshold for convergence.

Return $R_{\mathrm{best}}$ as the optimal solution.

### 2.3. MORIME

This study proposes a novel Multi-Objective Rime optimization algorithm (MORIME), which is based on the RIME algorithm and incorporates a stochastic inertial guidance mechanism, an adaptive target selection strategy, and an external non-dominated archive to effectively solve complex Multi-Objective optimization problems in truss structure design. Let $x^{*}$ denote a Pareto optimal solution such that there does not exist any $x^{\prime }\in \Omega$ satisfying $F\left(x^{\prime }\right)≻F\left(x^{*}\right)$, where ≻ indicates that $x^{\prime }$ is not dominated by $x^{*}$ in all objectives and is strictly better in at least one objective. An external archive $A_{t}$ is employed to store non-dominated solutions during the search process to ensure solution diversity and uniform distribution.

Step 1: Archive Update for Non-dominated Solutions

When a new candidate solution $x_{i}^{\prime }$ is generated, it is compared against the existing archive members $\left\{x_{1},x_{2},\ldots ,x_{n}\right\}$. If $x_{i}^{\prime }$ is dominated by any member of the archive, it is discarded. Otherwise, $x_{i}^{\prime }$ is added to the archive, and any solutions in the archive that are dominated by $x_{i}^{\prime }$ are removed. If the archive size exceeds the predefined limit $N_{\max}$, a density estimation strategy is employed to remove individuals from crowded regions. The neighbor count $N_{i}$ of each solution is used to calculate the selection probability:

(49) $$P_{i}=\frac{1}{N_{i}+1}$$

Roulette wheel selection is then used to choose a guidance target from under-explored regions of the archive. Where $P_{i}$ denotes the probability of selecting the *i*-th non-dominated solution as the guidance target, and $N_{i}$ represents the neighbor count used to estimate the local density around the solution.

Step 2: Position Update Using Inertial Mass and Target Guidance

MORIME incorporates a position update rule that combines inertial mass, gravitational interaction, and target guidance. The update equation in the *d*-th dimension for individual *i* is defined as follows:

(50) $$x_{i,d}^{t+1}=\vartheta \cdot \left[\omega \cdot x_{i,d}^{t}+\alpha \cdot \sum_{j=1}^{N}F_{ij,d}+\beta \cdot g_{d}\right]$$

In this formula, ϑ is the regulation factor that adjusts the balance between exploration and exploitation. The parameter ω represents the inertia weight and is linearly decreased over iterations, defined as follows:

(51) $$\omega =\omega_{\max}-\frac{t}{t_{\max}}\left(\omega_{\max}-\omega_{\min}\right)$$

The parameters α and β are the coefficients for gravitational attraction and target guidance, respectively. The term $F_{ij,d}$ represents the gravitational force exerted by individual *j* on individual *i* in dimension *d*, and is defined as follows:

(52) $$F_{ij,d}=\frac{ub_{d}-lb_{d}}{2}\cdot S\left(∥x_{j}^{d}-x_{i}^{d}∥\right)\cdot \frac{x_{j}^{d}-x_{i}^{d}}{∥x_{j}^{d}-x_{i}^{d}∥}$$

Here, $ub_{d}$ and $lb_{d}$ are the upper and lower bounds of variable *d*, respectively, $g_{d}$ is the selected global guidance target in dimension *d*, and S(·) denotes the social interaction function defined in Equation (52).

The social interaction function S(·) is defined as follows:

(53) $$S\left(r\right)=f_{0}\cdot e^{-r/\ell }-e^{-r}$$

where $f_{0}$ controls the strength of attraction and *ℓ* controls the interaction range. The values of $f_{0}$ and *ℓ* are chosen based on the problem’s characteristics to ensure effective interaction.

Step 3: Multi-Objective Evaluation and Ranking

All objective functions are normalized before aggregation. For the *k*-th objective of the *i*-th individual:

(54) $$f_{k}^{i}=\frac{f_{k}^{i}-\min\left(f_{k}\right)}{\max\left(f_{k}\right)-\min\left(f_{k}\right)},\;k=1,2,\ldots ,K$$

where $f_{k}^{i}$ denotes the normalized value of the *k*-th objective for the *i*-th individual, and *K* is the total number of objective functions. Then the multi-criteria evaluation index for each individual is computed as follows:

(55) $$MC_{i}=\sum_{k=1}^{K}f_{k}^{i}$$

where $MC_{i}$ represents the comprehensive evaluation score of the *i*-th individual obtained by aggregating all normalized objective values.

MORIME effectively combines inertia-based exploration, guided exploitation, and Pareto-based external archiving to maintain solution diversity and improve convergence performance. The regulation factor ϑ dynamically balances global exploration and local exploitation throughout the optimization process. Meanwhile, the adaptive target selection strategy guides the population toward high-quality non-dominated solutions, while the external archive preserves elite solutions and maintains diversity on the Pareto front. As the optimization proceeds, these mechanisms enable the population to gradually converge toward a stable Pareto-optimal region while reducing the likelihood of premature convergence. Although a rigorous mathematical proof of global convergence is beyond the scope of this study, the proposed optimization strategy provides a reliable and effective mechanism for determining the ensemble weights of the MF-PIV prediction framework.

## 3. Data Characterization

To prevent data leakage and respect the temporal order of the time series observations, the datasets were split chronologically rather than randomly. The first 80% of the sequential data points (in chronological order from earliest to latest recordings) were used for training the sub-models, the subsequent 16% for validation, and the final 4% for testing. This ensures that no future information is incorporated into the training process, providing a valid assessment of the model’s generalization in real-world scenarios.

The 80–16–4 partition was selected to balance model training, parameter validation, and independent performance evaluation. Specifically, the training set provides sufficient historical information for learning the nonlinear characteristics of wind speed data, while the validation set is used for parameter tuning and ensemble optimization. The remaining 4% is reserved as an independent test set to evaluate forecasting performance on unseen future data. Although the test set represents only 4% of the complete dataset, it still contains sufficient chronological observations to provide a reliable assessment of the model’s generalization ability.

This study employs wind speed datasets from two locations in Nanning, Guangxi, China. Specifically, Site A is located in Wuming and provides MF-based PIV high-frequency (HF) wind speed data recorded every 40 min. For the MF-based PIV high-frequency (HF) wind speed data, the maximum, minimum, and average wind speed values are calculated every 40 min, with the maximum value being the upper bound point, the minimum value being the lower bound point, and the average value being the middle value. There are a total of 4200 data points for each of the upper bound, lower bound, and middle values in the HF data. For the MF-based PIV low-frequency (LF) wind speed data, the maximum, minimum, and average wind speed values are calculated every 2 h, with the maximum value being the upper bound point, the minimum value being the lower bound point, and the average value being the middle value. There are a total of 1400 data points for each of the upper bound, lower bound, and middle values in the LF data.

Site B, situated in Xingning, provides MF-based PIV high-frequency (HF) wind speed data recorded every 30 min. For its HF data, the maximum, minimum, and average wind speed values are calculated every 30 min, with the maximum value being the upper bound point, the minimum value being the lower bound point, and the average value being the middle value. There are a total of 5600 data points for each of the upper bound, lower bound, and middle values in the HF data. The MF-based PIV low-frequency (LF) wind speed data are also calculated every 2 h, with a total of 1400 data points for each of the upper bound, lower bound, and middle values.

**Figure 2:**
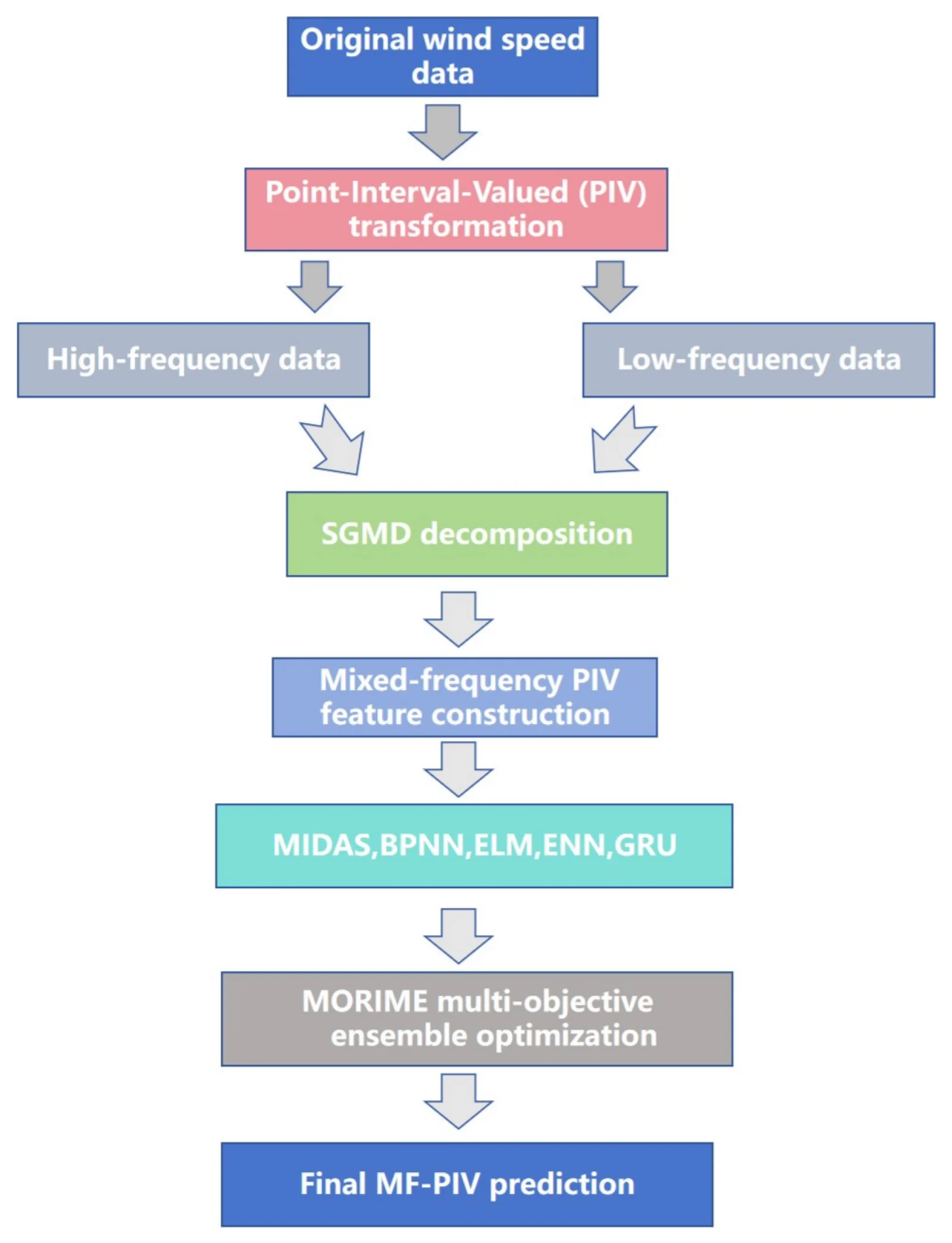
Overall framework of the proposed mixed-frequency point-interval-valued wind speed prediction system. The framework consists of three modules: (1) MF-PIV data preprocessing, (2) mixed-frequency prediction using multiple forecasting models, and (3) multi-objective ensemble optimization based on MORIME for final prediction.

At both locations, 80% of the dataset is employed to develop the sub-models, 16% of the data serves as the validation set, and 4% is allocated for testing the forecasting performance. These data are used to optimize sub-model parameters and construct the ensemble forecasting framework. The wind speed data used in this study were collected from April 2024 to May 2025, sourced from the Guangxi Meteorological Bureau. Wuming District (Site A) is located in a plain area, with relatively stable annual average wind speeds; Xingning District (Site B) is situated in a hilly region, where wind speed variations are more pronounced due to topographic uplift effects. Both areas experience a subtropical monsoon climate, but differences in terrain lead to distinct characteristics in their wind speed time series. Table 2 presents the performance comparison under different window size configurations. Table 3 provides detailed descriptions of the two datasets, while Figure 2 visualizes their statistical characteristics.

### Selection of High-Frequency and Low-Frequency Windows for Mixed-Frequency Point-Interval-Valued Data

According to the performance comparison of the system under different window combinations shown in Table 2, different combinations of HF and LF sliding window lengths were evaluated through preliminary experiments. The candidate window configurations were compared based on their prediction performance on the validation set using the proposed evaluation metrics, and the optimal configuration was selected according to the overall prediction accuracy and computational efficiency.

The following conclusions can be drawn: The combination of an HF window of 9 and an LF window of 3 is the optimal configuration. Its PIVMAE of 0.3771, PIVRMSE of 0.4547, and PIVMAPE of 4.10% are all at the lowest level, indicating that this setting best balances the utilization of historical information and noise suppression. When the window size is further increased, all error metrics increase significantly, and the computation time increases substantially, indicating that excessively long windows introduce redundant fluctuations and lead to overfitting. Therefore, all subsequent experiments in this study adopt HF = 9 and LF = 3 as the fixed sliding window settings. This choice ensures prediction accuracy while also taking into account the computational efficiency of the model.

## 4. Framework of the Wind Speed Prediction System

**Figure 3:**
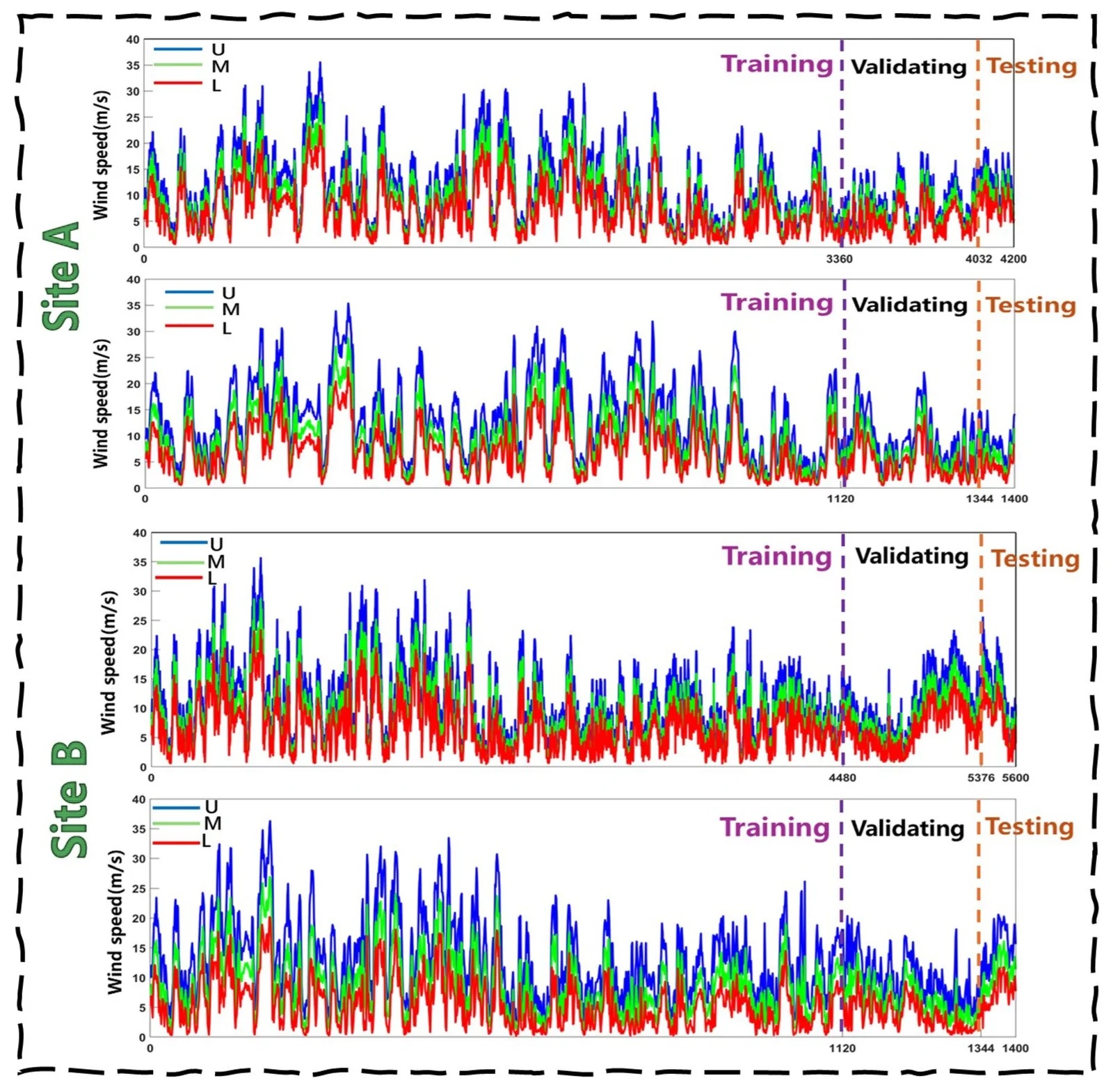
The study uses wind speed data from Site A and Site B.

Figure 3 illustrates the ensemble system, which consists of three main components: the PIV data preprocessing module for HF and LF data, the PIV prediction sub-module that combines both HF and LF data, and the ensemble module. The current investigation examines the HF and LF signals obtained from Wuming and Xingning monitoring stations. Both locations contain 1400 PIV wind speed measurements recorded at 2-h intervals: $PIV_{m}^{n}\left(1\mathrm{st}–1400\mathrm{th}\right)$, where n=U,M,L corresponds to the upper, middle, and lower thresholds, while *m* indicates Site A and Site B in sequence. The initial data subset $PIV_{m}\left(1\mathrm{st}–1120\mathrm{th}\right)$ is utilized for training five constituent models in the ensemble framework; the subsequent subset $PIV_{m}\left(1121\mathrm{st}–1344\mathrm{th}\right)$ acts as the validation set; the final subset $PIV_{m}\left(1345\mathrm{th}–1400\mathrm{th}\right)$ functions as the test set. The HF data collected at SiteA with a 40-min interval comprises a total of 12,600 PIV data points, which can be segmented into three parts. The HF data for the maximum value includes 4200 data points, the HF data for the median value includes 4200 data points, and the HF data for the minimum value also includes 4200 data points: $PIV_{A}\left(1\mathrm{st}–4200\mathrm{th}\right)$. The initial subset $PIV_{A}\left(1\mathrm{st}–3360\mathrm{th}\right)$ is utilized for the training phase; the subsequent subset $PIV_{A}\left(3361\mathrm{st}–4032\mathrm{th}\right)$ is employed for the validation phase and the final subset $PIV_{A}\left(4033\mathrm{st}–4200\mathrm{th}\right)$ is applied for the testing phase. The HF data collected at SiteB with a 30-min interval comprises a total of 16,800 PIV data points, which can also be segmented into three parts. The maximum value HF data includes 5600 points, the middle value HF data includes 5600 points, and the minimum value HF data includes 5600 points: $PIV_{B}\left(1\mathrm{st}–5600\mathrm{th}\right)$. The initial part $PIV_{B}\left(1\mathrm{st}–4480\mathrm{th}\right)$ is utilized for the training phase; the subsequent part $PIV_{B}\left(4481\mathrm{st}–5376\mathrm{th}\right)$ is employed for the validation phase and the final part $PIV_{B}\left(5377\mathrm{th}–5600\mathrm{th}\right)$ is applied for the testing phase. Figure 2 is the flowchart of the experimental framework.

Module 1 (Preprocessing of point-interval-valued (PIV) Data): This study adopts a range of decomposition and localization techniques tailored to the characteristics of diverse data types. Both the LF and HF PIV wind speed measurements are decomposed using the SGMD method.

Module 2 (Prediction with mixed-frequency (MF)-based point-interval-valued (PIV) Data): To enhance the accuracy of predicting MF-based PIV wind speed data, it is essential to employ multiple sub-models capable of capturing diverse temporal characteristics. Accordingly, five distinct prediction models are developed: one MIDAS model and four artificial intelligence-based approaches, namely BPNN, ELM, ENN, and GRU. Each model—MIDAS, BPNN, ENN, ELM, and GRU—utilizes combined HF and LF wind speed inputs to predict the upper, middle, and lower limits of the CF-derived PIV data. Furthermore, the GRU model is also employed on the reconstructed CF dataset to estimate the corresponding PIV upper, middle, and lower bounds.

Module 3 (Multi-Objective ensemble): To simultaneously consider the objectives of prediction accuracy and stability in wind speed prediction, determining the optimal weights for each sub-model proves to be a highly effective strategy. Based on this, an ensemble method grounded in the MORIME is adopted, with the inclusion of a tuning factor $\hat{\vartheta }$. This tuning factor serves to balance, fine-tune, or coordinate the relationships between different subsystems or objectives, and subsequently combines the prediction results derived from each sub-model based on PIV data.

To obtain the final results involving PIV data, a robust strategy is to jointly optimize the goals of prediction accuracy and stability by determining optimal sub-model weights. Therefore, a Multi-Objective ensemble module based on MORIME is designed to merge the prediction outcomes derived from each sub-model handling PIV data. The rationale behind selecting MORIME lies in its strong Multi-Objective optimization capabilities, high global search efficiency, and fast convergence speed, which make it particularly effective in addressing complex nonlinear challenges. A Multi-Objective ensemble module employing MORIME is designed to consolidate the prediction outputs from all constituent models with PIV inputs. The demonstrated superiority of MORIME in addressing the specified multicriteria optimization challenge outperforms conventional and state-of-the-art optimization methods, since no single algorithm can universally dominate across all problem domains. Within the proposed optimization framework, three weight categories are defined: upper-bound coefficients $\varphi_{m}^{i}$ with i∈{n∈N∣1≤n≤5}, middle-bound coefficients $\varphi_{m}^{k}$ where k∈{n∈N∣1≤n≤5}, and lower-bound coefficients $\varphi_{m}^{j}$ for j∈{n∈N∣1≤n≤5}. These weights are assigned to the five constituent models, where subscript *m* indicates Site A or Site B. A calibration parameter $\hat{\vartheta }$ is introduced to compute the final ensemble output. As an example, using the prediction outputs from the five sub-models for Site A, ${\hat{PIV}}_{A,i}^{U}$ (1121st–1344th), ${\hat{PIV}}_{A,k}^{M}$ (1121st–1344th), and ${\hat{PIV}}_{A,j}^{L}$ (1121st–1344th). The validation set’s fitted values for the upper, middle, and lower thresholds are computed using the following procedure:

(56) $$\begin{array}{cc} Set_{A}^{n}\left(1121\mathrm{th}–1344\mathrm{th}\right)= & \sum_{i=1}^{5}\varphi_{A}^{i}\times \left[{\hat{PIV}}_{A,i}^{U}\left(1121\mathrm{th}–1344\mathrm{th}\right)\right]\\ \; & +\sum_{k=1}^{5}\varphi_{A}^{k}\times \left[{\hat{PIV}}_{A,k}^{M}\left(1121\mathrm{th}–1344\mathrm{th}\right)\right]\\ \; & +\sum_{j=1}^{5}\varphi_{A}^{j}\times \left[{\hat{PIV}}_{A,j}^{L}\left(1121\mathrm{th}–1344\mathrm{th}\right)\right]+\hat{\vartheta } \end{array}$$

In Equation (56), n=U,M,L correspond to the upper bound, middle bound, and lower bound, respectively. The weights $\varphi_{A}^{i},\varphi_{A}^{k},\varphi_{A}^{j}$ are constrained such that $-2\leq \varphi_{A}^{i},\varphi_{A}^{k},\varphi_{A}^{j}\leq 2$. The parameter *u* is used to adjust the bias of each sub-model. It is noteworthy that the framework employs a unified approach to threshold prediction, collectively processing outputs for the upper, median, and lower bounds rather than relying on isolated single-threshold predictions. To determine the optimal weights and adjustment factor $\hat{\vartheta }$, this study aims to maximize prediction accuracy and stability, thereby formulating the following Multi-Objective optimization problem:

(57) $$\left\{\begin{array}{c} Obj_{A}^{1}=\mathrm{mean}\left(\left|\frac{PIV_{A}^{n}\left(1121\mathrm{th}–1344\mathrm{th}\right)-Set_{A}^{n}\left(1121\mathrm{th}–1344\mathrm{th}\right)}{PIV_{A}^{n}\left(1121\mathrm{th}–1344\mathrm{th}\right)}\right|\right)\\ Obj_{A}^{2}=\mathrm{std}\left[\frac{PIV_{A}^{n}\left(1121\mathrm{th}–1344\mathrm{th}\right)-Set_{A}^{n}\left(1121\mathrm{th}–1344\mathrm{th}\right)}{PIV_{A}^{n}\left(1121\mathrm{th}–1344\mathrm{th}\right)}\right] \end{array}\right.$$

Formula (57) can also be expressed as follows:

(58) $$\left\{\begin{array}{l} \left\{\varphi_{A}^{g}\right\}=\arg\min_{\left\{\varphi_{A}^{g}\right\}}\left\{Obj_{A}^{1},Obj_{A}^{2}\right\},\\ \arg\min_{\left\{\varphi_{A}^{g}\right\}}\left\{\begin{array}{c} \mathrm{mean}\;\left(\left|\frac{PIV_{A}^{n}\left(1121\mathrm{th}–1344\mathrm{th}\right)-Set_{A}^{n}\left(1121\mathrm{th}–1344\mathrm{th}\right)}{PIV_{A}^{n}\left(1121\mathrm{th}–1344\mathrm{th}\right)}\right|\right),\\ \mathrm{std}\;\left[PIV_{A}^{n}\left(1121\mathrm{th}–1344\mathrm{th}\right)-Set_{A}^{n}\left(1121\mathrm{th}–1344\mathrm{th}\right)\right] \end{array}\right.\\ \begin{array}{cc} s.t.\;Set_{A}^{n}\left(1121\mathrm{th}–1344\mathrm{th}\right)= & \sum_{i=1}^{5}\varphi_{A}^{i}\left[{\hat{PIV}}_{A,i}^{U}\left(1121\mathrm{th}–1344\mathrm{th}\right)\right]\\ \; & +\sum_{k=1}^{5}\varphi_{A}^{k}\left[{\hat{PIV}}_{A,k}^{M}\left(1121\mathrm{th}–1344\mathrm{th}\right)\right]\\ \; & +\sum_{j=1}^{5}\varphi_{A}^{j}\left[{\hat{PIV}}_{A,j}^{L}\left(1121\mathrm{th}–1344\mathrm{th}\right)\right]+\hat{\vartheta }. \end{array} \end{array}\right.$$

(59) $$\begin{array}{cc} Pre_{A}^{n}\left(1345\mathrm{th}–1400\mathrm{th}\right)= & \sum_{i=1}^{5}\varphi_{A}^{i}\times \left[{\hat{PIV}}_{A,i}^{U}\left(1345\mathrm{th}–1400\mathrm{th}\right)\right]\\ \; & +\sum_{k=1}^{5}\varphi_{A}^{k}\times \left[{\hat{PIV}}_{A,k}^{M}\left(1345\mathrm{th}–1400\mathrm{th}\right)\right]\\ \; & +\sum_{j=1}^{5}\varphi_{A}^{j}\times \left[{\hat{PIV}}_{A,j}^{L}\left(1345\mathrm{th}–1400\mathrm{th}\right)\right]+\hat{\vartheta } \end{array}$$

The MORIME algorithm is applied in this research to tackle the stated Multi-Objective optimization issue. The proposed methodology consists of the following steps: First, the parameters of the MORIME algorithm are initialized. Next, the initial best-performing solution is defined, and the iterative process is initiated to refine the positional information of the solution. Once the termination criteria are satisfied, the MORIME algorithm concludes, and the most favorable solution is presented as the final output of the multi-criteria optimization problem. Finally, an analysis and discussion of the experimental results based on PIV wind speed prediction are conducted.

### 4.1. Experimental Results and Analysis

This section presents the evaluation criteria, experimental design, and analysis of results. Six standard metrics are adopted to assess the prediction performance of each model: PIVMAE, PIVRMSE, PIVMAPE, PIVSSE, PIVU, and PIVARV. PIVMAE, PIVRMSE, PIVMAPE, and PIVSSE quantify the prediction accuracy of point-interval-valued forecasts from different perspectives, where lower values denote higher prediction accuracy. Specifically, PIVMAE measures the average prediction error, PIVRMSE places greater emphasis on larger prediction deviations, PIVMAPE evaluates the relative prediction error in percentage form, and PIVSSE reflects the accumulated squared prediction error. PIVARV and PIVU further evaluate the proposed PIV time series prediction approach: PIVARV measures the prediction error relative to the mean of the observed time series and reflects the overall prediction stability, while PIVU assesses the discrepancy between the reference model and the random walk benchmark. Values of PIVARV and PIVU closer to zero indicate superior predictive performance. Therefore, these six metrics provide a comprehensive evaluation of the proposed framework from the perspectives of prediction accuracy, relative error, accumulated error, and prediction stability. For a clear comparison across models, the metrics are summarized in Table 4.

To verify the prediction effectiveness of the system, 20 models are constructed and compared. Four experiments were devised to evaluate the prediction capability of the proposed system. Experiment 1 investigates the importance and effectiveness of the multi-criteria integrated framework by comparing six sub-models (Models 1–6) and assessing the influence of data denoising on the input data used for prediction. Experiment 2 assesses the value and essential role of the developed system enhanced with the MF prediction module by comparing it to ensemble methods based on sub-models (Models 7–11). Experiment 3 validates the improved performance of the integrated system combined with the MF-based PIV prediction approach by benchmarking it against alternative models (Models 12–16). Experiment 4 aims to highlight the significant advantage of the presented framework compared with other established models (Models 17–20), thereby emphasizing its benefits over commonly used prediction methods. The specifications of all twenty comparative models are detailed in Table 5.

### 4.2. Experiment 1: Comparison of Different Submodels

**Figure 4:**
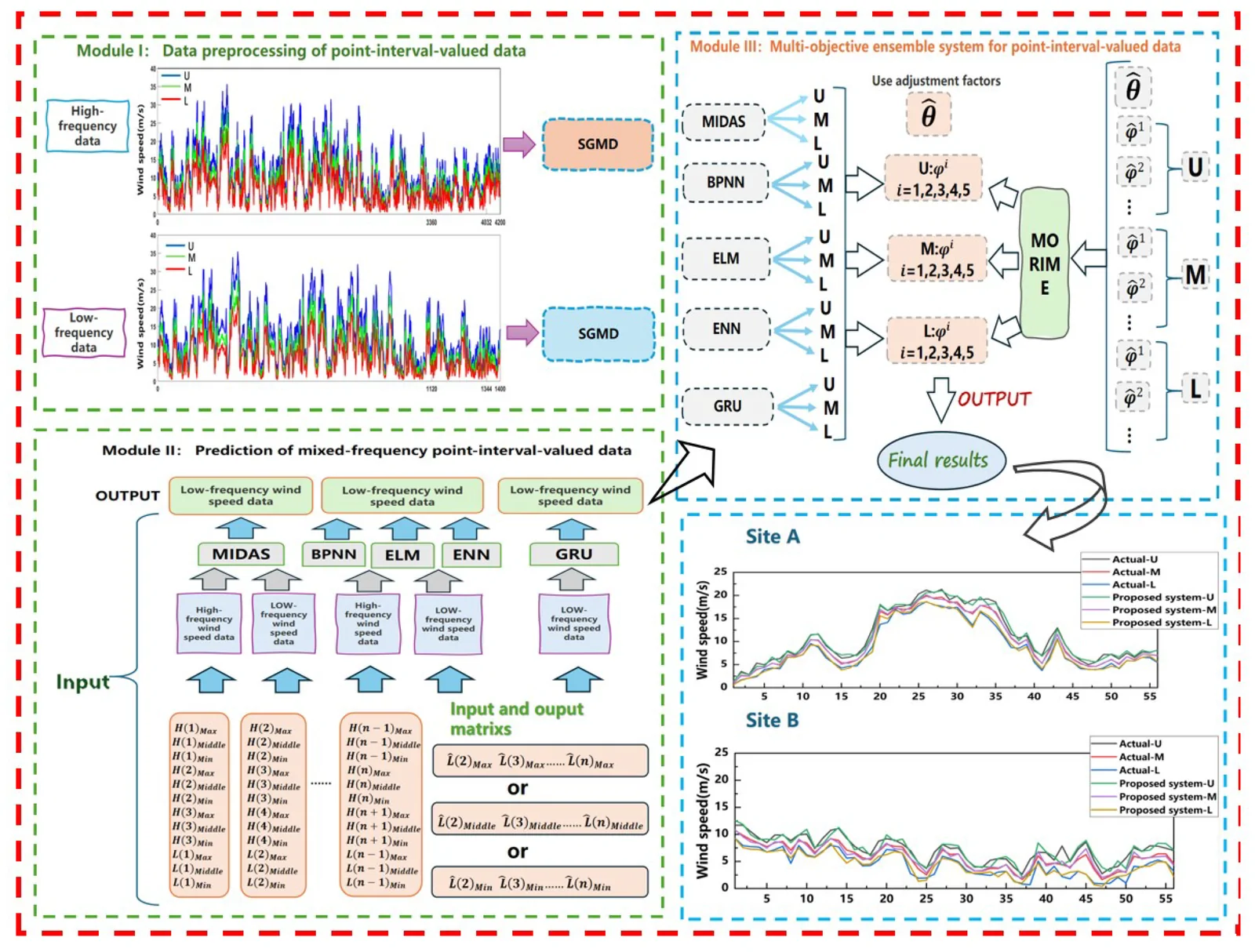
Three main modules of the experiment.

**Figure 5:**
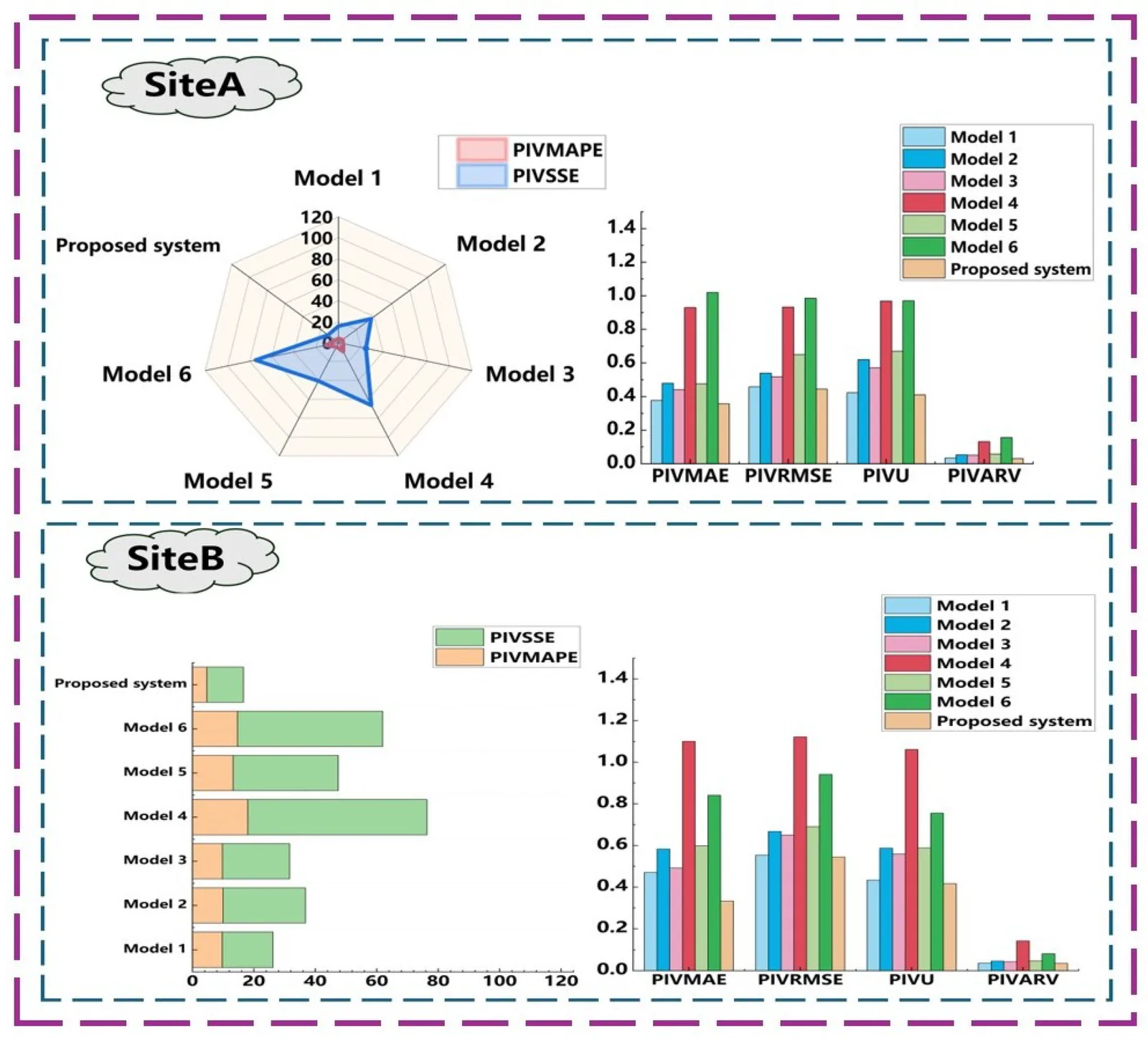
During data processing, the sub-models were compared with the prediction results of the proposed system.

The three main modules of the proposed framework are illustrated in Figure 4. As presented in Table 6 and Figure 5, a detailed evaluation of all experimental findings validates the benefits of the ensemble module, with support from its performance comparison involving the six sub-models. For instance, at Site A, the PIVMAE values for Models 1 through 6 are 0.3769, 0.4790, 0.4408, 0.9301, 0.4756, and 1.0201, respectively. Conversely, the proposed system achieves a markedly reduced PIVMAE, illustrating its enhanced prediction performance compared with the individual sub-models and underscoring the efficacy of the ensemble method.

At Site B, through a comparison of Model 6 with Models 1, 2, 3, and 5, it is evident that favorable outcomes have been obtained by employing SGMD to refine the data. Furthermore, the assessment of Model 4 against Models 1, 2, 3, and 5 reveals that utilizing MF data results in a significant enhancement of prediction accuracy. Additionally, the proposed system attains a PIVMAE value of 0.3329, the lowest across all assessed models, suggesting that it effectively leverages the advantages of every sub-model to deliver the most accurate prediction performance through integration of the PIV data from each sub-model.

**Experimental Analysis:** Experiment 1 aims to evaluate the effectiveness of the SGMD-based denoising strategy. As shown in the experimental results, the denoised wind speed series exhibit significantly reduced irregular fluctuations while preserving the essential temporal structures of the original signals. Compared with the raw data and other decomposition-based preprocessing methods, SGMD effectively separates noise-dominated components from informative signal components through symplectic geometric decomposition. This property enables the removal of high-frequency noise without excessively smoothing meaningful variations, which is crucial for wind speed data characterized by non-stationarity and strong volatility. The improved signal quality after SGMD preprocessing provides a more informative and stable input for subsequent prediction models.

**Note:** In this study, the proposed system attained the most favorable prediction results at both sites when compared with the individual sub-models. Moreover, the comparative analysis among the sub-models further underscores the benefits of employing MF data in enhancing prediction accuracy. At the same time, the denoising algorithm exhibited better performance during the data processing phase. Experimental results also showed that the denoised data outperformed the non-denoised data in terms of prediction performance, further validating the importance and efficiency of the denoising algorithm in wind speed prediction using PIV data.

### 4.3. Experiment 2: The Advantages and Importance of Mixed-Frequency Data

**Figure 6:**
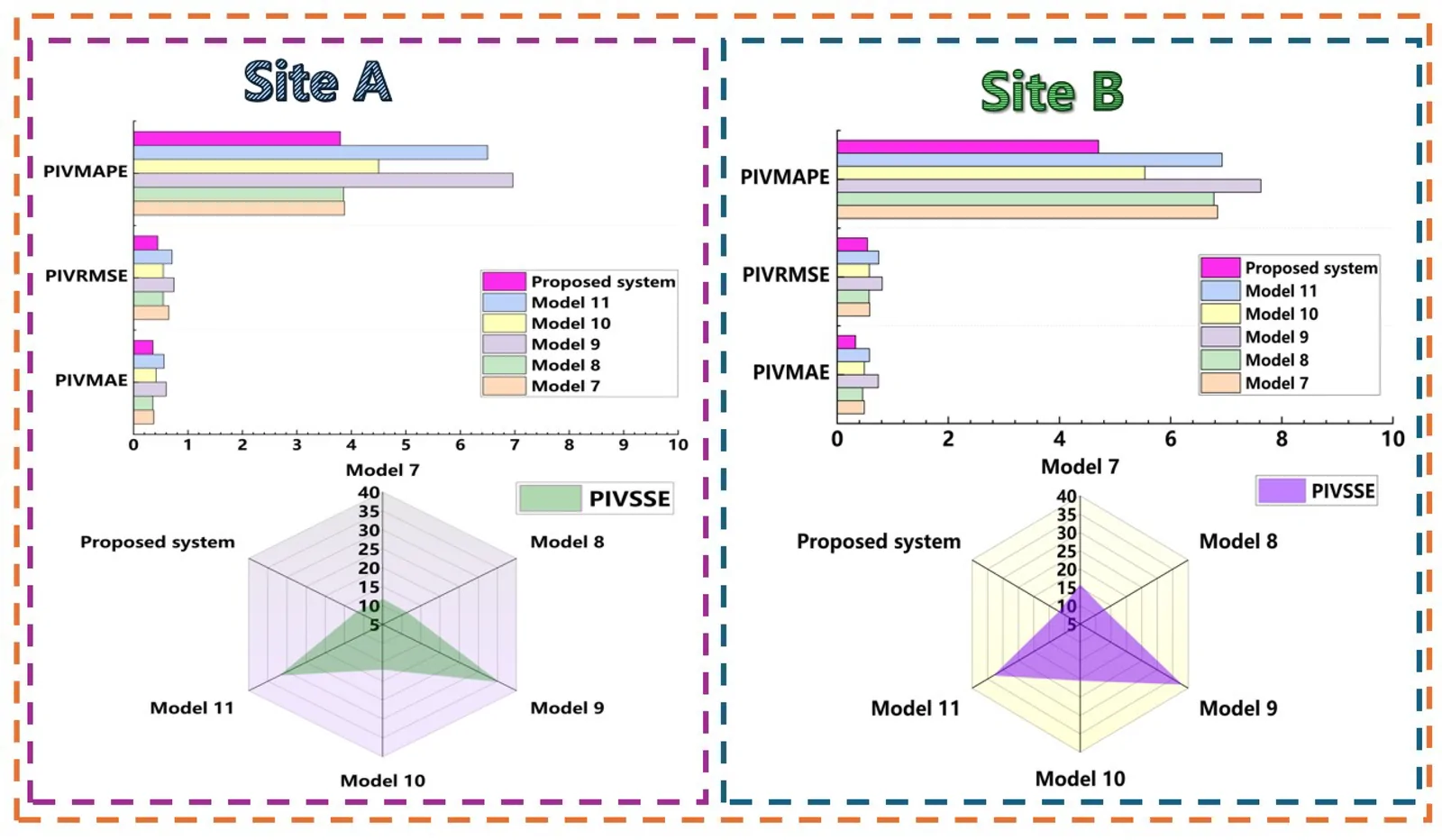
PIV prediction using MF data for multi-module results.

As presented in Table 7 and Figure 6, to confirm the advantages of the MF prediction module and further illustrate the importance and efficacy of utilizing MF data within the proposed system, Table 8 provides the comparative prediction results of five ensemble models (Models 7–11) constructed using various sub-model configurations. Model 7 and Model 8 incorporate BP, ELM, MIDAS, and GRU, while Models 9, 10, and 11 consist of GRU, BP, ELM, and ENN. Furthermore, Models 8, 10, and 11 utilize MF data, whereas Models 7 and 9 use CF data, thereby emphasizing the significance of incorporating MF data. For instance, at sites A and B, the evaluation metrics PIVMAE and PIVU are 0.3571, 0.4107 and 0.3329, 0.4171, respectively. Consequently, in terms of prediction performance, the proposed system demonstrates a clear advantage over other ensemble models, resulting in a substantial enhancement in prediction accuracy.

The experimental results of Model 7 and Model 9 indicate that the performance achieved using MF data is significantly superior to that obtained using CF data. This further confirms the advantage of MF in wind speed prediction. Additionally, by comparing Model 8 and Model 11, it can be found that when MF data is used, Model 8, which employs MIDAS, demonstrates significantly better prediction performance than Model 11, which does not employ MIDAS. This highlights the critical role of MIDAS in improving wind speed prediction accuracy. Furthermore, a comparative analysis of Model 7 versus Model 8 demonstrates that, compared with the GRU model, MIDAS demonstrates enhanced proficiency in processing MF data, highlighting the greater efficiency of MIDAS in managing such data for prediction purposes.

**Experimental Analysis:** Experiment 2 is designed to investigate the impact of MF information on wind speed prediction performance. The results clearly indicate that MF-based models consistently outperform their CF-based counterparts across all evaluation metrics. By employing the MIDAS mechanism, the proposed framework aligns HF predictors with LF targets without enforcing artificial aggregation. This alignment preserves fine-grained temporal information while maintaining consistency across frequencies. Consequently, the MF-based approach provides a more comprehensive representation of wind dynamics, leading to improved accuracy and interval reliability. These findings confirm that the observed performance gains are fundamentally driven by the effective exploitation of MF information rather than by model complexity alone.

**Note:** Through a detailed evaluation of the performance of various ensemble models, it can be inferred that the developed prediction system delivers optimal results across all sites, thereby further validating the significant improvements in both performance and efficacy of the ensemble system in leveraging MF prediction. Furthermore, Experiment II highlights the essential role of the MF multi-model ensemble prediction approach, which efficiently utilizes the strengths of each constituent model.

### 4.4. Experiment 3: Demonstrating the Superiority of the Mixed-Frequency-Based Point-Interval-Valued Prediction Ensemble System

**Figure 7:**
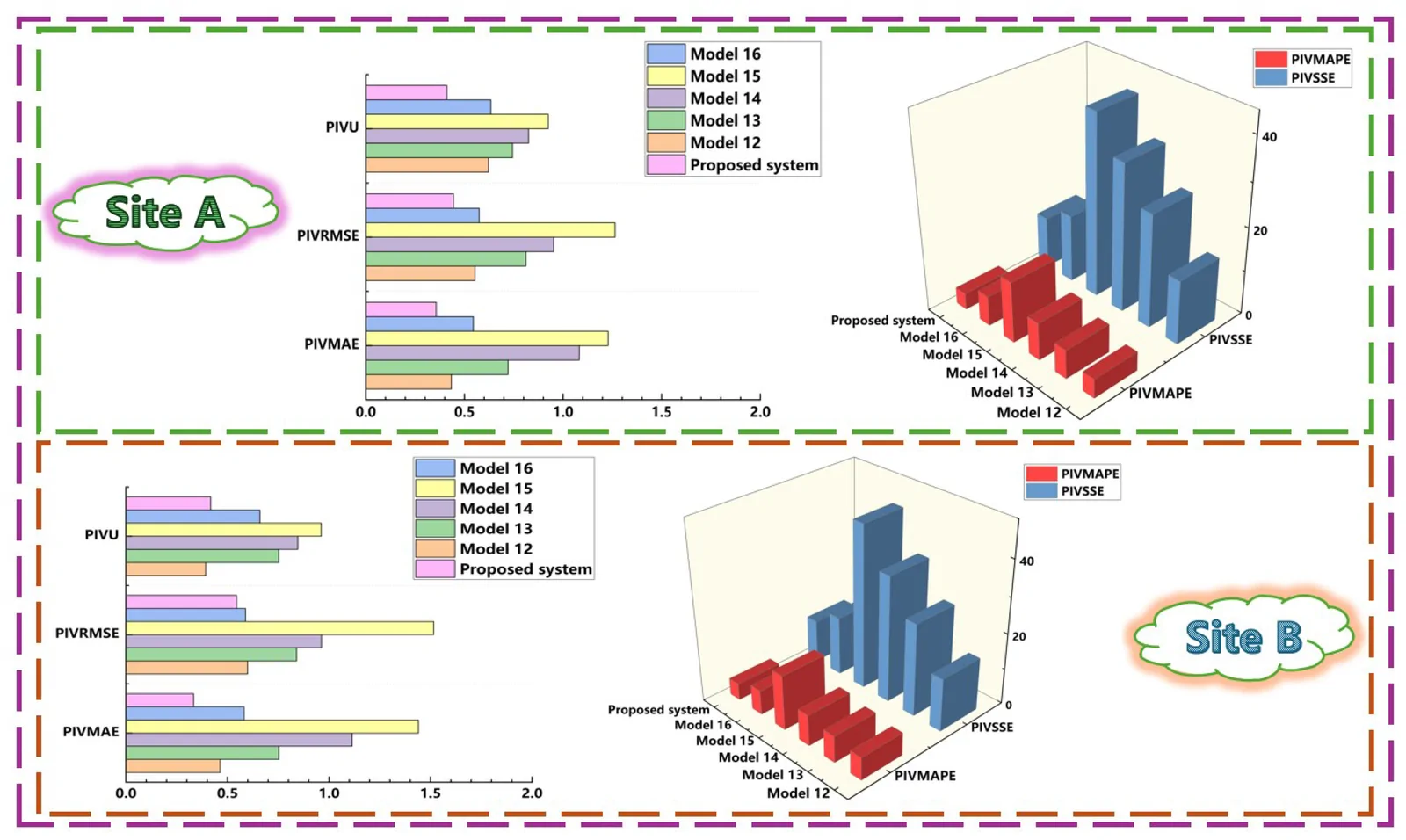
Performance comparison between the proposed ensemble prediction system and the constituent sub-models based on mixed-frequency point-interval-valued (MF-PIV) data. The figure illustrates the superiority of the proposed framework in terms of prediction accuracy and stability.

Five widely adopted prediction approaches (Models 12, 13, 14, 15, and 16) are utilized for comparative analysis, aiming to highlight the effectiveness of the proposed ensemble strategy, which incorporates PIV data. In particular, Models 12 and 14 are employed to contrast the most effective submodels derived from MF-based and common-frequency PIV combinations, respectively. Models 13 and 15 compare the worst submodel of the MF-based PIV combination with the worst submodel of the common-frequency PIV combination. Model 16 performs PIV ensemble prediction using only MIDAS. The specific experimental results are presented in Table 8 and Figure 7.

The assessment results across multiple metrics suggest that the proposed system, utilizing MF PIV data, achieves better prediction performance compared with current models. At Site A, the ensemble system achieves a PIVMAE value of 0.3571, compared with 0.4341 and 1.0821 for Models 12 and 14, respectively, indicating a reduced prediction error relative to these models. At Site B, the ensemble system’s PIVMAE value of 0.3329 remains the lowest among all evaluated models. These results strongly demonstrate the system’s superior performance in terms of the PIVMAE metric. Furthermore, the system records the lowest PIVRMSE values of 0.4447 at Site A and 0.5450 at Site B among all models, confirming not only enhanced prediction accuracy but also greater stability in its predictive outcomes.

Notably, the optimal sub-model incorporating MF-based PIV data (Model 12) outperforms the optimal sub-model utilizing CF-based PIV data (Model 14). Specifically, at Site A, Model 12 achieves a PIVMAE value of 0.4341, which is significantly lower than the 1.0821 of Model 14; while at Site B, the PIVMAE value of Model 12 is 0.4641, also lower than the 1.1140 of Model 15. These results confirm that the combination of MF-based PIV data significantly enhances prediction accuracy. Moreover, even the poorest-performing MF-based PIV sub-model (Model 13) outperforms the worst CF-based PIV sub-model (Model 15). For instance, at Site A, the PIVMAE value of Model 13 is 0.7210, markedly lower than the 1.2287 of Model 15; similarly, at Site B, the PIVMAE value of Model 13 is 0.7530, which is also lower than the 1.4405 of Model 15. These results further substantiate the superiority of the MF-based PIV approach in predicting wind speed.

**Experimental Analysis:** The results provide critical insights into the effectiveness of different multiobjective optimization algorithms in constructing MF-based PIV integrated prediction systems. By comparing the proposed MORIME-based system with ensemble models built upon four other established multiobjective algorithms (MODA, MOPSO, NSWOA, and MOMVO), this experiment aims to reveal the fundamental impact of optimizer selection on the final performance of the system. Specifically, the cooperative mechanism of soft rime exploration and hard rime penetration adopted by the MORIME algorithm enables it to dynamically balance global search and local exploitation, thereby locating the Pareto optimal region more effectively in the complex weight space. Simultaneously, its external archive and density-based selection strategy ensure that the final solution achieves a balanced trade-off among the multiple objectives of accuracy, stability, and reliability. In contrast, although the compared algorithms, such as the rapidly converging MOPSO or the exploration-emphasizing MOMVO, each have their own characteristics, their overall effectiveness is inferior to that of MORIME when dealing with the fine-grained trade-offs among multiple conflicting objectives in this task.

**Note:** Experiment III highlights the importance of incorporating MF-based PIV data, together with a multi-model ensemble approach that leverages the strengths belonging to every sub-model. The proposed system improves prediction accuracy while providing a more robust and consistent framework for wind speed prediction.

### 4.5. Experiment 4: The Proposed System Demonstrates Competitiveness Compared with Other Multi-Objective Combined Systems

**Figure 8:**
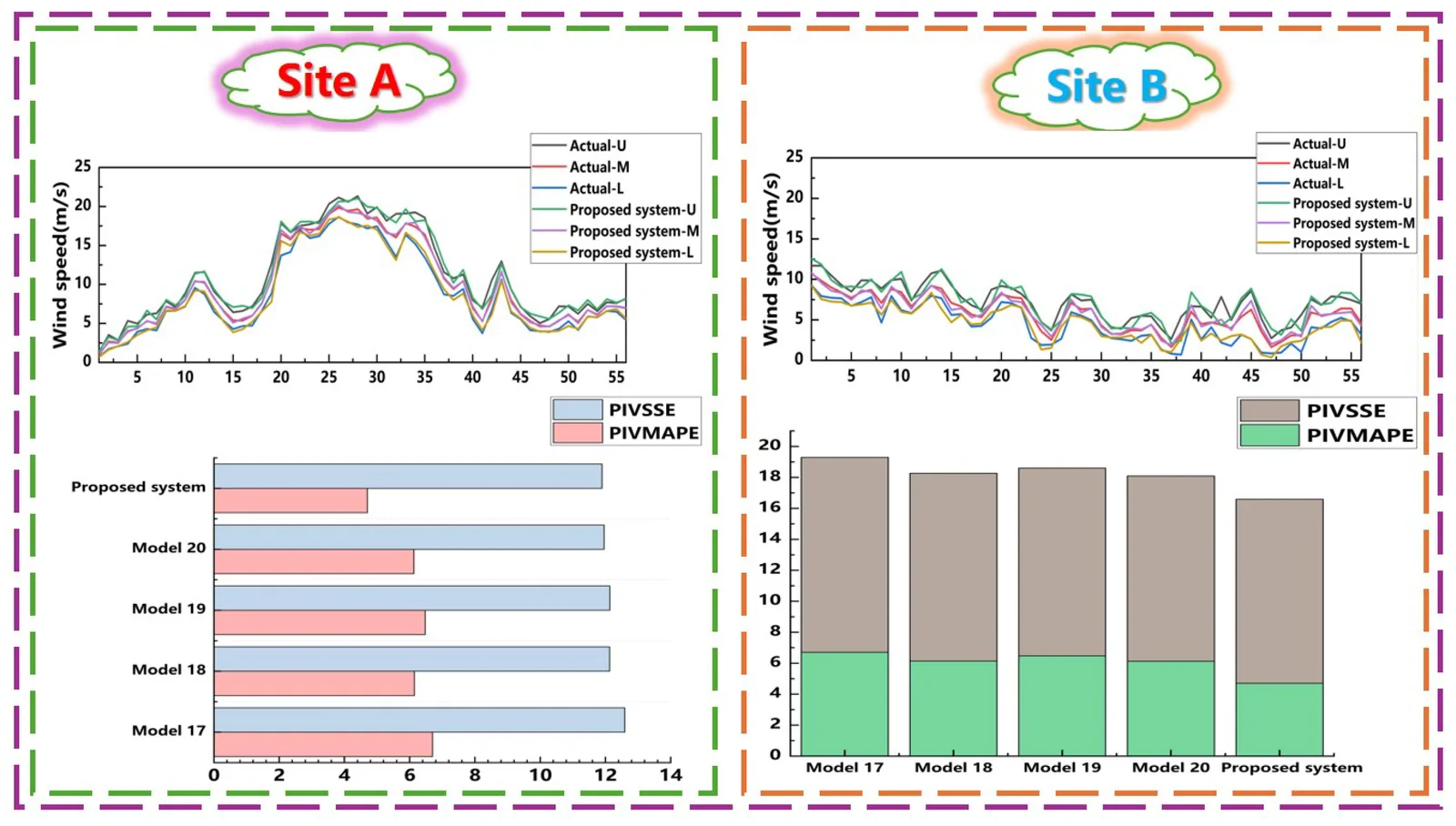
A comparison of the developed system with other Multi-Objective combined systems.

The Multi-Objective MORIME algorithm significantly enhances the search efficiency and solution diversity in complex Multi-Objective optimization tasks, making it especially suitable for optimizing truss structure designs [57]. As shown in Table 9 and Figure 8, although the ensemble system exhibits superior performance compared with constituent models, especially Models 17 [58], 18 [59], 19 [60], and 20 [61], it remains crucial to verify whether the proposed MORIME system outperforms other Multi-Objective ensemble methods when performing prediction with PIV data. The advantages of the incorporated optimization approach receive additional confirmation through benchmarking against four reference models (Models 17 to 20) that exclude adjustment factors. The corresponding results are detailed in Table 9.

As shown in Table 9 and Figure 8, for instance, at Site A, the PIVMAPE of the proposed system is 3.7984%, the smallest among all models at this location. By contrast, the PIVMAPE of Model 19 reaches 3.9175%, the largest across all evaluated models, highlighting the exceptional prediction precision of the proposed system.

At Site B, the PIVMAE for Model 17, Model 18, Model 19, and Model 20 are 0.3529, 0.3480, 0.3512, and 0.3381, respectively. The PIVMAE of the proposed system is 0.3329, the lowest at this site, further confirming the advantage of the proposed system in terms of stability. Moreover, this result suggests that the wind speed prediction results of the proposed system, which combines PIV data with adjustment factors, demonstrate a distinct level of superiority.

**Experimental Analysis:** The results provide critical insights into the effectiveness of different Multi-Objective optimization algorithms in constructing mixed-frequency point-interval-valued (MF-PIV) integrated prediction systems. By comparing the proposed MORIME-based system with ensemble models built upon four other established multi-objective algorithms (MODA, MOPSO, NSWOA, and MOMVO), this experiment aims to reveal the fundamental impact of optimizer selection on the final performance of the system. Specifically, the cooperative mechanism of soft-rime exploration and hard-rime penetration adopted by the MORIME algorithm enables it to dynamically balance global search and local exploitation, thereby locating the Pareto-optimal region more effectively in the complex weight space. Simultaneously, its external archive and density-based selection strategy ensure that the final solution achieves a balanced trade-off among the multiple objectives of accuracy, stability, and reliability. In contrast, although the compared algorithms, such as the rapidly converging MOPSO or the exploration-emphasizing MOMVO, each have their own characteristics, their overall effectiveness is inferior to that of MORIME when dealing with the fine-grained trade-offs among multiple conflicting objectives in this task.

**Note:** Whether at Site A or Site B, the proposed system outperforms across all evaluation metrics, delivering improved precision and consistency in wind speed prediction. This further confirms that the system, enhanced with adjustment factors, holds a significant advantage compared with the model without such factors.

## 5. Discussion I: Is the Multi-Objective Ensemble Module with Adjustment Factors Superior to Other Ensemble Methods $\hat{\vartheta }$?

**Figure 9:**
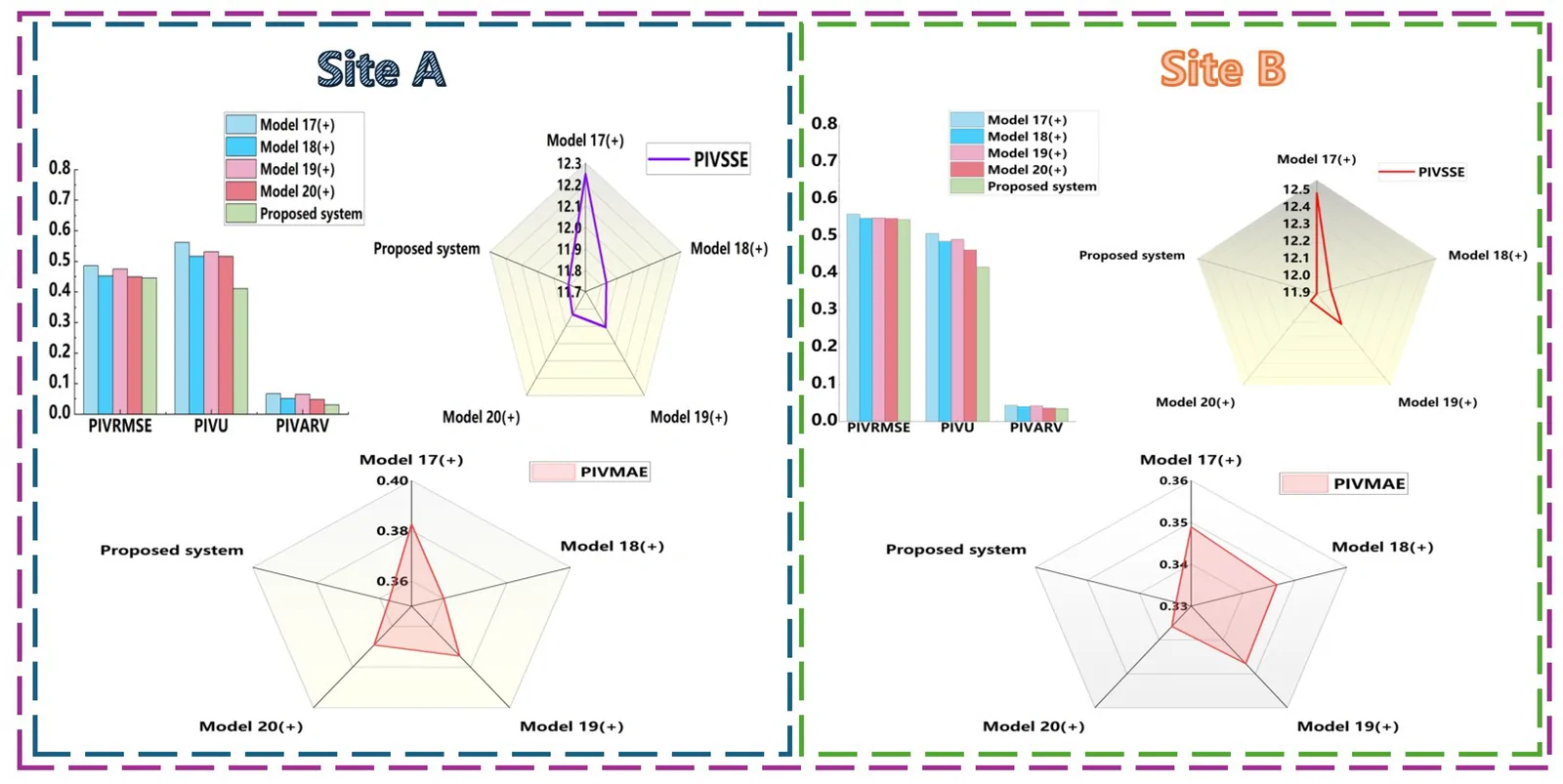
Comparison of predictive models with moderators and proposed system models.

In Experiment 4, it has been confirmed that the system developed with adjustment factors outperforms models lacking adjustment factors. A comparison of the proposed MORIME system with widely used models incorporating adjustment factors (models with adjustment factors are marked with (+)). As shown in Table 10 and Figure 9.

A review of the data in Table 9 and Table 10 shows that models incorporating adjustment factors, specifically Models 17, 18, 19, and 20, achieve better performance than those without adjustment factors. For instance, in Table 10, Model 19 records a PIVRMSE of 0.3745 and a PIVARV of 0.0652 at Site A. Meanwhile, in Table 10, Model 19’s PIVRMSE at Site A is 0.4797, and the PIVARV is 0.0682. These results further confirm that models with adjustment factors outperform those lacking them.

As presented in Table 10 and Figure 9, the ensemble system shows better performance compared with other ensemble models. Specifically, for location A, the PIVMAE achieved by this system is 0.3571, surpassing all comparative models, indicating that this approach yields reduced prediction errors. The PIVRMSE obtained by the system is 0.4447, outperforming all alternatives, further verifying its advantages regarding prediction stability. Additionally, the PIVSSE of this method is 11.8074, showing superior performance to all counterparts, indicating decreased prediction errors. The PIVU of our system is 0.4107, which similarly demonstrates the best results among all evaluated approaches, implying lower uncertainty in prediction. These results demonstrate that the ensemble system displays greater reliability and consistency in prediction tasks.

**Note:** The proposed system with adjustment factors demonstrates enhanced effectiveness relative to alternative benchmark models regarding prediction accuracy, stability. The presented model exhibits notable competitive strengths, potentially serving as a reliable approach for handling related tasks.

Despite the promising results, the proposed system has several limitations. The empirical validation is confined to two stations in Nanning, China, and the model’s generalizability to other geographic regions—particularly those with complex terrain or different climatic regimes—remains untested. In addition, the current forecasting horizon is limited to short-term predictions (aligned with the 2-h LF window), and the system’s scalability to larger datasets requires further optimization.

## 6. Discussion II: Model Efficiency Analysis

To evaluate the practical utility of the proposed system in real-world wind speed prediction tasks, Table 11 presents a comparison of computational time between the proposed system, five sub-models, and four other multi-objective ensemble models (Models 17–20). The results indicate that the proposed system has a runtime of 255.4972 s, which is longer than that of the single sub-models (57.45–98.75 s) but comparable to other multi-objective frameworks (218.5–252.1 s). Specifically, the proposed system is only about 17% slower than the fastest multi-objective model (Model 18) and shows a negligible difference (1.3%) compared with Model 19. Although the training time of the ensemble system is higher than that of individual sub-models, this increase is attributable to the simultaneous optimization of upper, middle, and lower bound weights, a structural requirement for PIV prediction that all multi-objective ensemble models must satisfy.

## 7. Discussion III: Statistical Significance Analysis

To further verify whether the performance improvement of the proposed framework is statistically significant, a Friedman test was conducted using the forecasting errors of all competing models on the testing dataset. The null hypothesis assumes that there is no significant difference among the compared models. The obtained *p*-value was less than 0.05, indicating that the null hypothesis can be rejected at the 95% confidence level.

## 8. Discussion IV: Computational Efficiency and Practical Deployment

The proposed MF-PIV prediction framework incorporates SGMD, MIDAS, multiple artificial intelligence prediction models (BPNN, ELM, ENN, and GRU), and the MORIME-based multi-objective ensemble optimization strategy within a unified architecture. Although the incorporation of multiple modules inevitably increases computational complexity compared with individual prediction models, each component serves a distinct purpose. Specifically, SGMD is employed to reduce noise and improve data quality, MIDAS effectively captures mixed-frequency information, the prediction models learn nonlinear relationships from different perspectives, and MORIME performs multi-objective ensemble optimization to enhance prediction accuracy and robustness. The coordinated operation of these modules enables the proposed framework to fully exploit both mixed-frequency information and point-interval-valued characteristics, thereby achieving superior prediction accuracy and robustness. Therefore, the increased computational complexity represents a reasonable trade-off for the substantial improvement in prediction performance.

From the perspective of practical deployment, it should be noted that the computationally intensive procedures, including signal decomposition, model training, and multi-objective optimization, are performed during the offline training stage. Once the optimal model parameters have been obtained, the online prediction stage only requires executing the trained prediction model, which significantly reduces the computational burden and satisfies the real-time requirements of short-term wind speed prediction. Therefore, the proposed framework remains suitable for practical wind energy management systems equipped with modern multi-core workstations or cloud computing platforms.

Furthermore, the proposed framework exhibits good scalability owing to its modular architecture. Each prediction model or optimization algorithm can be independently replaced or upgraded without affecting the overall prediction framework, allowing the proposed system to be easily adapted to different datasets, prediction horizons, and application scenarios. Future work will focus on developing lightweight prediction architectures, model compression techniques, and parallel computing strategies to further improve computational efficiency while maintaining high prediction accuracy.

Overall, although the proposed framework introduces additional computational overhead compared with individual prediction models, the significant improvements in prediction accuracy, robustness, and uncertainty representation justify the increased computational cost for practical short-term wind speed prediction.

### Computational Complexity Analysis

The computational complexity of the proposed MF-PIV prediction framework mainly originates from four stages: SGMD-based signal decomposition, mixed-frequency feature extraction using MIDAS, nonlinear prediction using multiple artificial intelligence models, and MORIME-based multi-objective ensemble optimization.

Assume that the input dataset contains *N* observations and the optimization algorithm performs *T* iterations with a population size of *P*. The computational complexity of SGMD is approximately O(N), the MIDAS regression requires O(Nd), the neural network models require approximately O(NE), and MORIME has a computational complexity of O(PT). Therefore, the overall computational complexity can be expressed as

O(N)+O(Nd)+O(NE)+O(PT),

which is dominated by the neural network training and multi-objective optimization stages.

The memory requirement increases approximately linearly with the dataset size and model complexity because the framework mainly stores decomposed sequences, neural network parameters, and the MORIME archive. As shown in Table 12, although the proposed framework requires a longer execution time than the competing models due to the integration of multiple prediction modules and optimization procedures, it achieves substantially higher prediction accuracy and robustness. Moreover, the computationally intensive procedures are performed during the offline training stage, while the online prediction stage only executes the trained model, making the proposed framework suitable for practical short-term wind speed prediction.

## 9. Conclusions

This study aims to improve the efficiency of wind speed prediction and overcome the limitations of existing prediction methods through the utilization of MF-based PIV data. To this end, a Multi-Objective ensemble system based on MF-based PIV combinations is proposed for wind speed prediction, aiming to enhance prediction accuracy and overall performance. The system consists of three primary modules: a PIV data preprocessing module for handling both HF and LF inputs, an MF-based PIV prediction module that incorporates HF and LF information, and a Multi-Objective ensemble system module. Specifically, the proposed ensemble prediction system offers the following advantages: (1) the development of a PIV prediction model; (2) the integration of MF data into the framework, constructing an innovative wind speed prediction ensemble system by coupling MF data with PIV features; (3) based on the characteristics of PIV data, this study proposes a novel data preprocessing strategy. This strategy employs the standard SGMD algorithm to jointly denoise both LF and HF wind speed components within the MF-PIV framework; (4) an improved Multi-Objective prediction system is proposed, combining PIV data with adjustment factors; (5) six evaluation indicators are proposed: PIVMAPE, PIVMAE, PIVRMSE, PIVU, PIVSSE, and PIVARV. Finally, a series of experiments are conducted to assess the prediction outcomes and compare them with other models, demonstrating the notable superiority of the proposed system in prediction performance. Experimental results show that the system achieves PIVMAE and PIVRMSE values of 0.3571 and 0.4447 at Site A, and 0.3329 and 0.5450 at Site B, respectively, outperforming all comparative models. Although the proposed system provides an effective and forward-looking approach for wind speed prediction and achieves significant improvements in prediction accuracy, some limitations remain and warrant further investigation. Specifically, this study focuses on the development of wind speed prediction models based on MF-based PIV data, considering only the influence of past wind speed information on prediction performance. Future studies may incorporate additional variables affecting wind speed changes to establish more comprehensive multi-factor prediction frameworks. Furthermore, from a practical perspective, the prediction method and system proposed in this study hold great potential for broader applications, including power demand prediction and securities investment prediction. In the future, the MF-PIV paradigm proposed in this study opens a clear path for subsequent exploration: first, integrating and benchmarking it with the continuously evolving latest deep learning models; second, based on this data foundation, exploring more diverse models to comprehensively enhance the performance boundaries of wind speed prediction. This system demonstrates good predictive performance at two stations in Nanning, but its extrapolation capability in complex terrain still requires further validation. Future work will consider incorporating multi-source data such as terrain elevation and humidity, and enhance the model’s adaptability to different geographical conditions through a transfer learning framework.

### 9.1. Experimental Environment

The algorithm development and numerical experiments in this study were conducted in the MATLAB R2022a environment. The hardware platform used is a workstation equipped with a 12th Gen Intel Core i5-12500H processor, 128 GB of RAM, and an NVIDIA GeForce RTX 4050 Laptop GPU. All optimization algorithms (e.g., MOPSO, MODA) were implemented in MATLAB, and the neural network models were constructed using the Deep Learning Toolbox.

### 9.2. Hyperparameter Settings

The hyperparameters of the BPNN, GRU, ELM, and ENN models were determined based on recommendations from the relevant literature and preliminary experiments. The final parameter settings were selected according to the prediction performance on the validation set. To ensure a fair comparison, the same hyperparameter settings were adopted throughout all comparative experiments. A comprehensive hyperparameter sensitivity analysis was beyond the scope of this study and will be considered in future work.

## Figures and Tables

**Table 1:** Summary of Prior Forecasting Models.

Citation	Researchers	Year	Modeling Strategy	Forecast Form and Frequency Type
[12]	Yan et al.	2012	Physical strategy	PV prediction (CF)
[13]	Mayer and Yang	2023	Physical strategy	PV prediction (CF)
[14]	Vahidi and Porté-Agel	2022	Physical strategy	PV prediction (CF)
[16]	Demolli et al.	2019	Statistical strategy	PV prediction (CF)
[15]	Hu, Wang, and Tao	2021	Hybrid strategy	PV prediction (CF)
[17]	Yan et al.	2023	Ensemble strategy	PV prediction (CF)
[19]	Chen et al.	2024	Ensemble strategy	PV prediction (MF)
[42]	Ma et al.	2024	Hybrid strategy	PV prediction (MF)
[21]	Yang et al.	2022	Ensemble strategy	PV prediction (MF)
[20]	An et al.	2024	Hybrid strategy	PV prediction (MF)
[43]	Tao et al.	2025	Hybrid strategy	PV prediction (MF)
[18]	Hu et al.	2025	Hybrid strategy	PV prediction (MF)
[22]	Jiang et al.	2023	Hybrid strategy	PV prediction (MF)
[23]	Li et al.	2025	Ensemble strategy	PV prediction (MF)
[24]	Tian and Hao	2020	Ensemble strategy	PV-to-IV prediction (CF)
[25]	Jiang et al.	2021	Ensemble strategy	PV-to-IV prediction (CF)
[26]	Li et al.	2023	Ensemble strategy	PV to IV prediction (CF)
[27]	Zeng et al.	2024	Ensemble strategy	PV-to-IV prediction (CF)
[31]	Wang et al.	2023	Hybrid strategy	PV-to-IV prediction (CF)
[29]	Wang et al.	2024	Hybrid strategy	PV-to-IV prediction (CF)
[30]	Wang et al.	2025	Hybrid strategy	PV-to-IV prediction (CF)
[28]	Yang et al.	2023	Ensemble strategy	PV-to-IV prediction (MF)
[32]	Sun et al.	2024	Ensemble strategy	IV prediction (CF)
[33]	Chang et al.	2024	Ensemble strategy	IV prediction (CF)
[34]	Liu et al.	2024	Ensemble strategy	IV prediction (CF)
[35]	Hou et al.	2024	Ensemble strategy	IV prediction (CF)
[36]	Zheng et al.	2024	Hybrid strategy	IV prediction (CF)
[37]	Yang et al.	2025	Ensemble strategy	IV prediction (CF)
[38]	Zhu et al.	2022	Ensemble strategy	IV prediction (CF)
[39]	Tang et al.	2023	Ensemble strategy	IV prediction (CF)
[40]	Hao et al.	2024	Ensemble strategy	IV prediction (CF)
[41]	Liu et al.	2025	Ensemble strategy	IV prediction (MF)

**Table 2:** Performance comparison under different window size configurations.

HF Window	LF Window	PIVMAE	PIVRMSE	PIVMAPE (%)	Computation Time (s)
9	3	0.3771	0.4547	4.10	185.2
12	6	0.3891	0.4876	4.21	203.7
36	9	0.4213	0.5124	4.56	255.5
48	24	0.3952	0.5120	4.71	342.8
96	36	0.4789	0.5235	5.05	489.3

**Table 3:** Data description.

Site	Type	Dataset	Numbers				Statistical Indicator			
				**Max**	**Min**	**Median**	**Mean**	**Std**	**Skewness**	**Kurtosis**
SiteA	$L_{h}$	All datasets	1400	25.2060	1.1611	8.3435	9.3213	5.0733	0.6055	2.6275
		Training	1120	25.2060	1.1611	9.4657	9.9564	5.2495	0.4424	2.3948
		Validating	224	15.9991	1.2448	6.4106	6.9060	3.4640	0.6434	2.5516
		Testing	56	10.9930	2.1823	6.1627	6.2810	1.9961	0.2815	3.0284
	$L_{m}$	All datasets	1400	23.7093	0.6667	6.9831	7.8605	4.7834	0.6763	2.7592
		Training	1120	23.7093	0.6667	8.1941	8.4529	4.9474	0.5133	2.5157
		Validating	224	14.8318	0.7201	5.1122	5.6245	3.4852	0.8499	3.0103
		Testing	56	9.4283	1.2265	4.9581	4.9562	1.8422	0.3871	3.0590
	$L_{l}$	All datasets	1400	22.9425	0.2052	5.6776	6.4070	4.5583	0.7734	2.9313
		Training	1120	22.9425	0.2052	6.5294	6.9377	4.7207	0.6203	2.6648
		Validating	224	14.2660	0.2326	3.5472	4.4547	3.2487	0.9844	3.3552
		Testing	56	8.3867	0.6056	3.6516	3.6027	1.7499	0.5919	3.2177
SiteA	$H_{h}$	All datasets	4200	25.2060	0.4651	7.7271	8.4428	4.8016	0.6880	2.9172
		Training	3360	25.2060	0.4651	8.2019	8.8934	5.0520	0.5674	2.5981
		Validating	672	13.2842	0.7919	5.6245	5.6245	2.8606	0.3976	2.3404
		Testing	168	14.1206	5.0764	9.1936	9.1936	2.0720	0.2039	2.4201
	$H_{m}$	All datasets	4200	23.8147	0.4305	7.0246	7.6938	4.6640	0.7177	2.9643
		Training	3360	23.8147	0.4305	7.4045	8.1315	4.9055	0.5988	2.6412
		Validating	672	12.6042	0.6079	4.9609	5.2968	2.7937	2.6412	2.4007
		Testing	168	13.3766	4.1381	8.4848	8.5284	1.9737	0.1371	2.5056
	$H_{l}$	All datasets	4200	23.1452	0.2052	6.3310	6.9525	4.5641	0.7457	3.0142
		Training	3360	23.1452	0.2052	6.7479	7.3783	4.7947	0.6321	2.6950
		Validating	672	12.4425	0.2326	4.2150	4.6072	2.7801	0.5582	2.4847
		Testing	168	12.7850	3.3152	7.8938	7.8173	1.9447	0.1119	2.6567
SiteB	$L_{h}$	All datasets	1400	24.8486	0.6314	8.2577	9.0469	4.5751	0.7566	3.2095
		Training	1120	24.8486	0.6314	7.2511	7.9379	4.8091	0.7773	3.2191
		Validating	224	16.2414	0.9228	7.0761	7.3700	2.7755	0.5271	2.0673
		Testing	56	17.8158	1.1655	10.0576	9.4389	2.2833	−0.3072	2.3145
	$L_{m}$	All datasets	1400	22.4608	0.7484	6.5130	7.1754	4.1800	0.8386	3.4068
		Training	1120	22.4608	0.7484	6.7743	7.4569	4.4066	0.7615	3.1098
		Validating	224	11.7634	1.3297	5.0002	5.2686	2.4226	0.4002	2.3463
		Testing	56	13.4429	4.1436	9.0066	9.1723	2.2552	0.0798	2.0171
	$L_{l}$	All datasets	1400	20.2075	0.2052	4.5697	5.3107	3.9226	0.9621	3.6573
		Training	1120	20.2075	0.2052	4.7355	5.5195	4.1273	0.9107	3.4002
		Validating	224	11.0137	0.3769	3.1679	3.6921	2.3420	0.5877	2.5462
		Testing	56	11.7597	1.4789	7.4197	7.6103	2.3706	0.0508	2.3252
SiteB	$H_{h}$	All datasets	5600	25.2060	0.6720	7.5497	8.1690	4.4156	0.7069	3.1597
		Training	4480	25.2060	0.6720	7.5048	8.2099	4.5663	0.7686	3.1956
		Validating	896	16.5231	0.9336	7.2345	7.5913	3.6251	0.2470	2.0615
		Testing	224	18.4316	1.2547	10.2464	9.6607	3.7949	−0.2926	2.3463
	$H_{m}$	All datasets	5600	23.8663	0.4409	6.8182	7.3556	4.2702	0.7249	3.1966
		Training	4480	23.8663	0.4409	6.7474	7.3654	4.4045	0.7996	3.2749
		Validating	896	15.9413	0.7555	6.6335	6.9020	3.5607	0.2622	2.0895
		Testing	224	16.4149	0.9988	9.4560	8.9742	3.7201	−0.3071	2.2685
	$H_{l}$	All datasets	5600	23.2772	0.2052	6.0752	6.5446	4.1705	0.7442	3.2247
		Training	4480	23.2772	0.2052	5.9359	6.5253	4.2900	0.8320	3.3451
		Validating	896	15.3684	0.3769	6.0811	6.2110	3.5340	0.2745	2.1265
		Testing	224	15.1012	0.3524	8.9989	8.2661	3.6721	−0.3154	2.1956

Note: $L_{h}$, $L_{m}$, and $L_{l}$ represent the upper, middle, and lower thresholds of the low-frequency PIV time series. Correspondingly, $H_{h}$, $H_{m}$, and $H_{l}$ indicate the upper, middle, and lower bounds of the HF-based PIV time series.

**Table 4:** Evaluation metrics.

Metric	Equation
PIVMAE	$\frac{1}{3n}\sum_{k=1}^{n}\left[\left|z_{l}\left(k\right)-{\hat{z}}_{l}\left(k\right)\right|+\left|z_{m}\left(k\right)-{\hat{z}}_{m}\left(k\right)\right|+\left|z_{h}\left(k\right)-{\hat{z}}_{h}\left(k\right)\right|\right]$
PIVRMSE	$\frac{1}{3}\left[\sqrt{\frac{1}{n}\sum_{k=1}^{n}{\left(z_{l}\left(k\right)-{\hat{z}}_{l}\left(k\right)\right)}^{2}}+\sqrt{\frac{1}{n}\sum_{k=1}^{n}{\left(z_{m}\left(k\right)-{\hat{z}}_{m}\left(k\right)\right)}^{2}}+\sqrt{\frac{1}{n}\sum_{k=1}^{n}{\left(z_{h}\left(k\right)-{\hat{z}}_{h}\left(k\right)\right)}^{2}}\right]$
PIVMAPE	$\frac{1}{3n}\sum_{k=1}^{n}\left[\left|\frac{z_{l}\left(k\right)-{\hat{z}}_{l}\left(k\right)}{z_{l}\left(k\right)}\right|+\left|\frac{z_{m}\left(k\right)-{\hat{z}}_{m}\left(k\right)}{z_{m}\left(k\right)}\right|+\left|\frac{z_{h}\left(k\right)-{\hat{z}}_{h}\left(k\right)}{z_{h}\left(k\right)}\right|\right]\times 100\%$
PIVSSE	$\frac{1}{3}\sum_{k=1}^{n}\left[{\left(z_{l}\left(k\right)-{\hat{z}}_{l}\left(k\right)\right)}^{2}+{\left(z_{m}\left(k\right)-{\hat{z}}_{m}\left(k\right)\right)}^{2}+{\left(z_{h}\left(k\right)-{\hat{z}}_{h}\left(k\right)\right)}^{2}\right]$
PIVU	$\sqrt{\frac{\sum_{k=1}^{n}{\left(z_{k+1}^{u}-{\hat{z}}_{k+1}^{u}\right)}^{2}+\sum_{k=1}^{n}{\left(z_{k+1}^{m}-{\hat{z}}_{k+1}^{m}\right)}^{2}+\sum_{k=1}^{n}{\left(z_{k+1}^{l}-{\hat{z}}_{k+1}^{l}\right)}^{2}}{\sum_{k=1}^{n}{\left(z_{k+1}^{u}-z_{k}^{u}\right)}^{2}+\sum_{k=1}^{n}{\left(z_{k+1}^{m}-z_{k}^{m}\right)}^{2}+\sum_{k=1}^{n}{\left(z_{k+1}^{l}-z_{k}^{l}\right)}^{2}}}$
PIVARV	$\frac{\sum_{k=1}^{n}{\left(z_{k+1}^{u}-{\hat{z}}_{k+1}^{u}\right)}^{2}+\sum_{k=1}^{n}{\left(z_{k+1}^{m}-{\hat{z}}_{k+1}^{m}\right)}^{2}+\sum_{k=1}^{n}{\left(z_{k+1}^{l}-{\hat{z}}_{k+1}^{l}\right)}^{2}}{\sum_{k=1}^{n}{\left(z_{k+1}^{u}-z_{k}^{u}\right)}^{2}+\sum_{k=1}^{n}{\left(z_{k+1}^{m}-z_{k}^{m}\right)}^{2}+\sum_{k=1}^{n}{\left(z_{k+1}^{l}-z_{k}^{l}\right)}^{2}}$

Note: *z* and $\hat{z}$ represent the actual and predicted PIV, respectively; *n* denotes the number of samples; *k* denotes the wind speed at the *k*-th time point; *u* and *h* represent the upper bound of PIV; *l* represents the lower bound of PIV and *m* represents the middle bound of PIV.

**Table 5:** Descriptions of the comparative models.

Explanation	Model
MIDAS model incorporating MF data following the SGMD data preprocessing algorithm	Model 1
BP model incorporating MF data following the SGMD data preprocessing algorithm	Model 2
ELM model incorporating MF data following the SGMD data preprocessing algorithm	Model 3
GRU model incorporating CF data following the SGMD data preprocessing algorithm	Model 4
ENN model incorporating MF data following the SGMD data preprocessing algorithm	Model 5
The BP model incorporating MF data preprocessing algorithms for unprocessed data	Model 6
Ensemble model assembled from BP and ELM incorporating MF data and GRU incorporating CF data	Model 7
Ensemble model assembled from ELM, BP, and MIDAS incorporating MF data	Model 8
Ensemble model assembled from GRU incorporating CF data and BP and ENN incorporating CF data	Model 9
Ensemble model assembled from BP and ENN incorporating MF data	Model 10
Ensemble model assembled from ELM and BP incorporating CF data	Model 11
The prediction results obtained from the best sub-model, along with the PIV prediction of MF data, encompass the lower-bound, middle-bound, and upper-bound	Model 12
The prediction results obtained from the worst sub-model, together with the PIV prediction of MF data, include the lower-bound, middle-bound, and upper-bound	Model 13
Prediction result derived from the best sub-model, combined with the ensemble of PIV estimates for CF data, including the lower-bound, middle-bound, and upper-bound	Model 14
Prediction results derived from the worst sub-model, aggregation of PIV predictions for CF data, including the lower-bound, middle-bound, and upper-bound	Model 15
The ensemble model of MF data PIV based on MIDAS, including the upper-bound, middle-bound, and lower-bound	Model 16
Development of a Multi-Objective model incorporating MODA	Model 17
Development of a Multi-Objective model incorporating MOPSO	Model 18
Development of a Multi-Objective model incorporating NSWOA	Model 19
Development of a Multi-Objective model incorporating MOMVO	Model 20

**Table 6:** Prediction performance of the examined constituent models and the ensemble system.

Site	Model	PIVMAE	PIVRMSE	PIVMAPE (%)	PIVSSE	PIVU	PIVARV
Site A	Model 1	0.3769	0.4575	4.0522	15.8371	0.4231	0.0339
	Model 2	0.4790	0.5397	4.4003	36.5366	0.6201	0.0531
	Model 3	0.4408	0.5180	4.3066	24.7195	0.5721	0.0510
	Model 4	0.9301	0.9327	9.5300	66.2903	0.9686	0.1314
	Model 5	0.4756	0.6497	4.4253	40.5866	0.6701	0.0574
	Model 6	1.0201	0.9861	11.4506	74.6035	0.9701	0.1556
	**Proposed system**	**0.3571**	**0.4447**	**3.7984**	**11.8074**	**0.4107**	**0.0306**
Site B	Model 1	0.4707	0.5542	9.6900	16.5174	0.4337	0.0367
	Model 2	0.5831	0.6672	9.9758	26.7836	0.5872	0.0459
	Model 3	0.4919	0.6501	9.7859	21.8333	0.5593	0.0430
	Model 4	1.1004	1.1213	18.0166	58.3420	1.0612	0.1416
	Model 5	0.5990	0.6902	13.2500	34.2278	0.5887	0.0467
	Model 6	0.8410	0.9410	14.6630	47.2381	0.7552	0.0823
	**Proposed system**	**0.3329**	**0.5450**	**4.7028**	**11.8932**	**0.4171**	**0.0350**

**Table 7:** Prediction results obtained from the ensemble model and prediction system, compared with those from alternative sub-models using MF and PIV data.

Site	Model	PIVMAE	PIVRMSE	PIVMAPE (%)	PIVSSE	PIVU	PIVARV
Site A	Model 7	0.3701	0.6454	3.8736	11.7435	0.4912	0.0480
	Model 8	0.3538	0.5451	3.8579	11.4268	0.4615	0.0430
	Model 9	0.6003	0.7409	6.9656	35.4649	0.7408	0.0830
	Model 10	0.4132	0.5454	4.5017	16.9262	0.5241	0.0537
	Model 11	0.5600	0.7069	6.5017	32.0212	0.6279	0.0743
	**Proposed system**	**0.3571**	**0.4447**	**3.7984**	**11.8074**	**0.4107**	**0.0306**
Site B	Model 7	0.4895	0.5858	6.8441	15.8325	0.6093	0.0583
	Model 8	0.4576	0.5733	6.7776	12.8720	0.5982	0.0570
	Model 9	0.7390	0.8109	7.6248	38.0445	0.7953	0.0838
	Model 10	0.4899	0.5784	5.5347	20.4912	0.5233	0.0591
	Model 11	0.5806	0.7476	6.9257	33.0980	0.6522	0.0750
	**Proposed system**	**0.3329**	**0.5450**	**4.7028**	**11.8932**	**0.4171**	**0.0350**

**Table 8:** Comparison of MF-based PIV combined prediction models.

Site	Model	PIVMAE	PIVRMSE	PIVMAPE (%)	PIVSSE	PIVU	PIVARV
Site A	Model 12	0.4341	0.5536	4.4104	14.6707	0.6217	0.0445
	Model 13	0.7210	0.8119	6.6133	25.9600	0.7442	0.0683
	Model 14	1.0821	0.9524	8.6044	33.8953	0.8251	0.0751
	Model 15	1.2287	1.2637	14.0509	41.8019	0.9247	0.1050
	Model 16	0.5452	0.5748	6.8524	15.6057	0.6341	0.0537
	**Proposed system**	**0.3571**	**0.4447**	**3.7984**	**11.8074**	**0.4107**	**0.0306**
Site B	Model 12	0.4641	0.5985	6.4367	14.8641	0.3932	0.0487
	Model 13	0.7530	0.8400	7.5037	26.0176	0.7527	0.0693
	Model 14	1.1140	0.9630	8.7158	35.2536	0.8460	0.0800
	Model 15	1.4405	1.5156	15.3701	45.6028	0.9617	0.1474
	Model 16	0.5813	0.5885	6.8681	16.5244	0.6592	0.0557
	**Proposed system**	**0.3329**	**0.5450**	**4.7028**	**11.8932**	**0.4171**	**0.0350**

**Table 9:** Comparison of different Multi-Objective combinations.

Site	Model	PIVMAE	PIVRMSE	PIVMAPE (%)	PIVSSE	PIVU	PIVARV
Site A	Model 17	0.3844	0.4874	3.8886	12.7777	0.5760	0.0716
	Model 18	0.3686	0.4544	3.8361	12.6545	0.5220	0.0514
	Model 19	0.3758	0.4797	3.9175	12.6796	0.5570	0.0682
	Model 20	0.3768	0.4538	3.8268	12.4914	0.5489	0.0448
	**Proposed system**	**0.3571**	**0.4447**	**3.7984**	**11.8074**	**0.4107**	**0.0306**
Site B	Model 17	0.3529	0.5620	6.7028	12.5932	0.5171	0.0450
	Model 18	0.3480	0.5498	6.1425	12.1260	0.5133	0.0439
	Model 19	0.3512	0.5508	6.4738	12.1323	0.5246	0.0440
	Model 20	0.3381	0.5492	6.1312	11.9575	0.5102	0.0368
	**Proposed system**	**0.3329**	**0.5450**	**4.7028**	**11.8932**	**0.4171**	**0.0350**

**Table 10:** Comparison of different Multi-Objective combinations with adjustment factors

Site	Model	PIVMAE	PIVRMSE	PIVMAPE (%)	PIVSSE	PIVU	PIVARV
Site A	Model 17(+)	0.3827	0.4854	3.8710	12.2500	0.5617	0.0671
	Model 18(+)	0.3601	0.4516	3.8094	11.8314	0.5161	0.0506
	Model 19(+)	0.3745	0.4750	3.8475	11.9044	0.5310	0.0652
	Model 20(+)	0.3691	0.4491	3.8063	11.8306	0.5156	0.0471
	**Proposed system**	**0.3571**	**0.4447**	**3.7984**	**11.8074**	**0.4107**	**0.0306**
Site B	Model 17(+)	0.3489	0.5601	6.5110	12.4802	0.5077	0.0437
	Model 18(+)	0.3465	0.5486	6.1277	11.9701	0.4861	0.0400
	Model 19(+)	0.3470	0.5498	6.4607	12.1148	0.4920	0.0425
	Model 20(+)	0.3360	0.5483	6.1270	11.9475	0.4630	0.0361
	**Proposed system**	**0.3329**	**0.5450**	**4.7028**	**11.8932**	**0.4171**	**0.0350**

**Table 11:** Compare the time of calculation.

Model	Time (s)
SGMD-GRU	85.4687
SGMD-ELM	57.4501
SGMD-ENN	78.2434
SGMD-BPNN	72.1535
SGMD-MIDAS	98.7548
Model 17	247.3
Model 18	218.5
Model 19	252.1
Model 20	236.8
**Proposed system**	**255.4972**

**Table 12:** Execution time comparison of representative prediction models.

Model	Training Time (s)	Prediction Time (ms)	Relative Cost
MIDAS	2.41	2.3	Low
BPNN	5.87	1.8	Medium
ELM	1.92	1.2	Low
ENN	8.65	2.6	Medium
GRU	12.74	3.1	High
MOPSO-based Ensemble	24.18	4.2	High
MODA-based Ensemble	26.47	4.5	High
Proposed MF-PIV (MORIME)	29.83	4.8	High

## Data Availability

The data supporting the findings of this study are available from the corresponding author upon reasonable request, subject to permission from the data provider.

## Abbreviations

The following abbreviations are used in this manuscript:

PIV	Point-Interval-Valued
PV	Point-Valued
IV	Interval-Valued
CF	Common-Frequency
MF	Mixed-Frequency
HF	High-Frequency
LF	Low-Frequency
SGMD	Symplectic Geometric Mode Decomposition
BPNN	Backpropagation Neural Network Architecture
ELM	Extreme Learning Machine
ENN	Elman Neural Network
GRU	Gated Recurrent Unit
MIDAS	Mixed Data Sampling
MORIME	Multi-Objective Rime Ice Meta-heuristic algorithm
MOMVO	Multi-Objective Multi-Verse Optimizer
MOTSA	Multi-Objective Tunicate Swarm Algorithm
MODA	Multi-Objective Dragonfly Algorithm
NSWOA	Multi-Objective Whale Optimization Algorithm
MOPSO	Multi-Objective Particle Swarm Optimization Algorithm
PIVMAPE	Point-Interval-Valued Mean Absolute Percentage Error
PIVMAE	Point-Interval-Valued Mean Absolute Error
PIVRMSE	Point-Interval-Valued Root Mean Square Error
PIVU	Point-Interval-Valued Theil’s U Statistics
PIVSSE	Point-Interval-Valued Sum of Squared Error
PIVARV	Point-Interval-Valued Mean Relative Variability
